# Altered methylation of imprinted genes in neuroblastoma: implications for prognostic refinement

**DOI:** 10.1186/s12967-024-05634-5

**Published:** 2024-08-31

**Authors:** Medha Suman, Maja Löfgren, Susanne Fransson, Jewahri Idris Yousuf, Johanna Svensson, Anna Djos, Tommy Martinsson, Per Kogner, Teresia Kling, Helena Carén

**Affiliations:** 1 Sahlgrenska Center for Cancer Research, Department of Medical Biochemistry and Cell Biology, Institute of Biomedicine, Sahlgrenska Academy, University of Gothenburg, Medicinaregatan 1F, 405 30 Gothenburg, Sweden; 2 Department of Laboratory Medicine, Institute of Biomedicine, Sahlgrenska Academy, University of Gothenburg, Gothenburg, Sweden; 3 Childhood Cancer Research Unit, Women’s, and Children’s Health, Karolinska Institutet, Stockholm, Sweden

**Keywords:** Neuroblastoma, Genomic imprinting, DNA methylation, Copy number alterations

## Abstract

**Background:**

Neuroblastoma (NB) is a complex disease, and the current understanding of NB biology is limited. Deregulation in genomic imprinting is a common event in malignancy. Since imprinted genes play crucial roles in early fetal growth and development, their role in NB pathogenesis could be suggested.

**Methods:**

We examined alterations in DNA methylation patterns of 369 NB tumours at 49 imprinted differentially methylated regions (DMRs) and assessed its association with overall survival probabilities and selected clinical and genomic features of the tumours. In addition, an integrated analysis of DNA methylation and allele-specific copy number alterations (CNAs) was performed, to understand the correlation between the two molecular events.

**Results:**

Several imprinted regions with aberrant methylation patterns in NB were identified. Regions that underwent loss of methylation in > 30% of NB samples were DMRs annotated to the genes *NDN*, *SNRPN*, *IGF2*, *MAGEL2* and *HTR5A* and regions with gain of methylation were *NNAT*, *RB1* and *GPR1*. Methylation alterations at six of the 49 imprinted DMRs were statistically significantly associated with reduced overall survival: *MIR886*, *RB1*, *NNAT*/*BLCAP*, *MAGEL2*, *MKRN3* and *INPP5F*. *RB1*, *NNAT/BLCAP* and *MKRN3* were further able to stratify low-risk NB tumours i.e. tumours that lacked *MYCN* amplification and 11q deletion into risk groups. Methylation alterations at *NNAT/BLCAP, MAGEL2* and *MIR886* predicted risk independently of *MYCN* amplification or 11q deletion and age at diagnosis. Investigation of the allele-specific CNAs demonstrated that the imprinted regions that displayed most alterations in NB tumours harbor true epigenetic changes and are not result of the underlying CNAs.

**Conclusions:**

Aberrant methylation in imprinted regions is frequently occurring in NB tumours and several of these regions have independent prognostic value. Thus, these could serve as potentially important clinical epigenetic markers to identify individuals with adverse prognosis. Incorporation of methylation status of these regions together with the established risk predictors may further refine the prognostication of NB patients.

**Supplementary Information:**

The online version contains supplementary material available at 10.1186/s12967-024-05634-5.

## Background

Neuroblastoma (NB) is an aggressive childhood malignancy that originates from immature cells of the neural crest with primary tumours commonly arising in adrenal medulla or in paraspinal sympathetic ganglia. NB accounts for 8–10% of all childhood cancer cases and is the most common form of cancer that affects infants (median age at diagnosis 17 months) [1]. NB is a heterogeneous disease with diverse biological and clinical features, ranging from low-risk localized cases that show spontaneous regression without any treatment to high-risk NB (HR-NB) cases featuring widely metastatic tumours with frequent adverse outcome [2]. The outcome for children with HR-NB, constituting almost half of all NB cases remains poor (5-year overall survival rate < 50%) despite an intense multi-modal treatment regime. The outcome is even worse for patients with relapsed or refractory disease (survival rate < 10%), as the cure for relapsed cases is limited [3]. Although advances in treatment have been made, management of HR-NB patients is especially challenging given the young age of patients, disease heterogeneity, treatment resistance and organ toxicity. Furthermore, survivors of this aggressive multi-modal therapy still remain at risk for long-term complications that can lead to excess morbidity, premature mortality, and impaired quality of life.

The genomic landscape of NB is well-studied with only a few genes shown to be altered recurrently by somatic genetic events, where mutations in *ALK*, encoding a receptor tyrosine kinase, being the most frequent; ~ 10% at primary diagnosis [4–6] and in 20–43% of patients with relapsed or refractory NB [7–9]. Instead of events affecting single genes, genetic alterations in NB predominantly harbour copy number alterations (CNAs) where HR-NB are associated with *MYCN* amplification or 11q deletion, and a higher rate of other segmental chromosomal aberrations including 1p deletion and 17q gain [10, 11]. However, the mechanisms that triggers the accumulation of chromosomal alterations leading to NB at such an early age is still not clear, and only a few specific genes in regions affected by recurrent segmental aberrations are identified to have clinical and therapeutic impact (e.g. *ALK*, *TERT*, *ATRX* or *CDKN2A/B*) [12–15].

Impairment of the normal sympathoadrenal differentiation is critical for NB development, meaning that NB retains embryonic features, which ultimately promote intratumour heterogeneity and resistance to therapy [16]. Molecular clock analysis suggests that NB starts developing as early as the first trimester of pregnancy where early genomic instability and prolonged evolution is associated with aggressive disease and, that aneuploidy is present at an early stage [17]. Furthermore, recent studies on foetal developmental trajectories and tumoural transcriptional cell state indicate that low-risk NB mainly associates to highly differentiated sympathoblasts whereas HR-NB associates to earlier developmental time points [18, 19]. This further supports that subversion of the normal differentiation process is critical for NB development and that deregulation of genes important for embryonal growth and development could provide an early hit that may lead to accumulation of further downstream molecular events.

Genomic imprinting is a mechanism in which a small group of genes (~ 1%) are expressed in a parent-of-origin specific manner [20]. To date, around 150 imprinted genes have been reported in humans of which many have major roles in early foetal growth and development. The mono-allelic expression of imprinted genes is regulated by a differential methylation pattern at respective parental allele that are inherited and maintained throughout the somatic development. Any aberration (genetic or epigenetic) in these regions may lead to loss of imprinting (LOI), which is associated with a range of human disorders. Some of the well-known examples are Angelman Syndrome (functional loss of the maternally active *UBE3A* allele), Prader-Willi Syndrome (loss of genes under control of the paternally active *SNURF*-*SNRPN* promoter) and Beckwith-Wiedemann Syndrome (LOI within the chromosome 11p15.5 region) [21]. LOI has also been reported as a common and early event in several adult tumours including colorectal cancer, liver cancer and in leukemia [22–24], with imprinting aberrations showing potential prognostic [24] and diagnostic value [25]. These findings suggest the importance of the imprinting mechanism and its role in tumour initiation and progression.

Enhancing the knowledge about NB pathogenesis is crucial, as there is an urgent need to develop an effective and less toxic way of treating the disease. This could possibly be achieved by a deeper understanding of the disease pathology and the identification of molecular alterations and pathways in NB pathogenesis that can be targeted. The notable lack of identified driving events in some NB tumours imply an additional layer of dysregulation and thus, a critical question is which other aberrations subvert normal development, cause NB, or add to heterogenous tumour behaviour. As imprinting have been implicated in tumour initiation and progression in other malignancies, we studied the DNA methylation landscape of NB with a focus on imprinted regions to identify regions with altered methylation patterns. Identification of dysregulated imprinted loci in NB tumour samples will enhance our understanding of the disease biology and potentially lead to the identification of molecular markers for novel treatment approaches.

## Materials and methods

### Study samples

The samples included in this study were obtained from the Therapeutically Applicable Research to Generate Effective Treatments (TARGET) and German Neuroblastoma Trial, merged as discovery set and a local cohort of NB tumours, used as validation set as described below.

### Public datasets

NB cases in TARGET were diagnosed between the years 1995–2011 and the age at diagnosis ranged from 0 to 20 years (median age 3 years). The tumours were diagnosed as stage 1 (8%), stage 2 (0.5%), stage 3 (3%) and stage 4/4 s (89%) according to the International Neuroblastoma Staging System (INSS). The tumours were mainly undifferentiated or poorly differentiated (79%) and most cases fell into the high-risk group (79%) according to the Children’s Oncology Group (COG) risk classification. *MYCN* amplification was seen in 25% of the cases and 11q deletion was seen in 38% of the cases. There were 93 deaths recorded during follow-up (median [IQR]: 7 [0–15] years). In the German Neuroblastoma Trial, cases were diagnosed between 1997 and 2004 and have been previously described [26]. Most of the cases in this cohort were diagnosed as stage 4/4 s tumours (73%), while the remaining were diagnosed as stage 3 (9%), stage 2 (9%) and stage 1 tumour (9%). The tumours were grouped as high-risk (56%), intermediate-risk (7%) and low-risk (37%). Amplification of *MYCN* was observed in 33% of cases while 27% had 11q deletion.

DNA methylation data (IDAT files) for primary NB (n = 213) samples was obtained from the TARGET-NB project (Study Accession: phs000467). For the German Neuroblastoma Trial (NB97 and NB2004), DNA methylation data was also obtained for 105 NB cases from Gene Expression Omnibus (GEO) GSE73515. For both the public datasets, genome-wide DNA methylation was assessed using the Infinium HumanMethylation450 BeadChip. Data from the two studies were merged and treated as the discovery set. For samples in the TARGET dataset, single nucleotide polymorphism (SNP) array data generated on three different Illumina platforms; HumanHap550, Human610-Quad and HumanOmniExpress from tumour and matching blood were downloaded from GSE131189 for 122/207 (59%) matching cases. Clinical and genomic data of the study participants are presented in Table 1.

**Table 1:** Clinical and pathological features of the study participants in the discovery and validation sets

Sample characteristics	Discovery set (n = 284)		Validation set (n = 85)
	TARGET NB (n = 185)	German Neuroblastoma Trial (n = 99)	Local NB Cohort (n = 85)
Median age at diagnosis (years), interquartile range	3.0 [25%; 1.7]	Missing	2.0 [25%, 0.9]
< 1.5 years (n, %) ≥ 1.5 years (n, %)	37 (20) 148 (80)	57 (58) 42 (42)	32 (38) 53 (62)
Gender (n, %)			
Male Female	113 (61) 72 (39)	Missing	33 (39) 52 (61)
Tumour stage (n, %), INSS			
Stage 1 Stage 2 Stage 3 Stage 4 Stage 4 s Unknown	14 (8) 1 (0.5) 6 (3) 146 (79) 18 (10) –	9 (9) 9 (9) 9 (9) 72 (54) 19 (19) –	2 (2) 8 (9) 13 (15) 33 (39) 3 (4) 26 (31)
Tumour grade (n, %)			
Differentiating Undifferentiated or poorly differentiated Unknown	10 (5) 146 (79) 29 (16)	Missing	Missing
COG Risk group (n, %)			
High risk Intermediate risk Low risk	147 (79) 9 (5) 29 (16)	55 (56) 7 (7) 37 (37)	Missing
MYCN status (n, %)			
Amplified Not amplified Unknown	47 (25) 138 (75) –	33 (33) 66 (67) –	28 (33) 56 (66) 1 (1)
11q deletion (n, %)			
11q deleted 11q normal Whole chromosome 11 gain Whole chromosome 11 loss Unknown	70 (38) 114 (62) – – 1 (0.5)	27 (27) 48 (48) 1 (1) 23 (23) –	20 (23) 64 (75) – – 1 (1.1)

*TARGET* therapeutically applicable research to generate effective treatments, *NB* neuroblastoma, *INSS* international neuroblastoma staging system, *COG* Children’s Oncology Group

### Validation cohort

The local cohort used as a validation set in this study included 85 NB tumours from a local cohort diagnosed between 1986 and 2023. Age of diagnosis ranged from 0 to 19 years (median age 2 years). Similar to the discovery set, most of the cases in the local cohort were higher stage tumours with 41% of tumours diagnosed as stage 4/4 s, 15% as stage 3, 9% stage 2 and 7% as stage 1. Tumour stage was unknown for 31% of the NB cases. A total of 34 deaths were recorded in the local cohort. Amplification of *MYCN* was observed in 32% of the cases while 11q deletion was observed in 23% of the cases in the local cohort. The clinical and pathological characteristics of the study participants are summarized in Table 1.

DNA methylation data for cases in the local cohort was assessed on three different methylation arrays: Illumina Infinium HumanMethylation450 (HM450K) array (n = 49) [27], EPIC array (n = 32) and EPIC array v2.0 (n = 4).

For investigation of normal tissue, healthy foetal adrenal gland tissue (n = 11) was used as controls and methylation data (Normalized methylation levels, beta-value) were obtained from Gene Expression Omnibus (GEO) GSE56515, n = 9 and GSE54719, n = 2. Methylation data from samples with maternal uniparental diploidy (mUPD) (n = 1) and paternal UPD (pUPD) (n = 2), used for visualization was obtained from GSE103738.

### Sample exclusion based on the copy number alteration profile

We used publicly available data from the samples included in the TARGET dataset (n = 216) and the German Neuroblastoma Trial (n = 105) as discovery set and a local cohort of samples as validation set (n = 97). Manual inspection of CNA plots was performed and only cases with distinct change at segmental alterations were kept in order to retain samples with increased likelihood for higher degree of neoplastic cells. After manual inspection of CNA-profiles, 26/213 cases from TARGET, 6/105 cases from the German Trial study and 12/97 cases from the local cohort were removed with suspicion of low tumour cell content and the reduced cohort sizes of n = 185, n = 99 and n = 85 for respective cohorts were used for further analyses. Copy number alteration plots using the methylation array data were generated using the R package conumee [28].

### Data preprocessing and normalization

#### Methylation array data

Methylation data for samples in the discovery set was preprocessed and normalized together to account for potential batch effects between the datasets. Raw methylation intensity files (IDATs) were imported into the R computing environment and data was pre-processed and normalised, using normalisation method “noobBMIQ” using the R package ChAMP [29]. Data quality was first accessed using parameters including detection P-value and bead count, which were calculated for every CpG position in every sample. Probes with an average detection P-value of > 0.01 were considered unreliable and were removed from further analysis. CpG probes that aligned to multiple sites and with a bead count of < 3 were also removed. Samples with failed p-value of > 0.05 were removed from further analysis. After data pre-processing and normalization, 284 out of 287 cases and a total of 438,729 CpG sites remained for further analysis. Beta-values (ratio of the methylated probe intensity and the sum of methylated and unmethylated probe intensity) were calculated for all the NB cases. For the validation dataset, data from different methylation array platforms were pre-processed and normalised separately and beta-values at common probes were merged. The same thresholds for data filtering were applied for the validation set. After data pre-processing 85 cases remained for further analysis.

#### SNP array data

Processed SNP array data (TXT files) from tumour and matching blood were downloaded from GSE131189 for 105/185 (57%) matching cases in the TARGET dataset. SNP data was generated using three different Illumina chips: HumanHap550, Human610-Quad and HumanOmniExpress with a common set of 316,210 probes.

### Imprinting regions analysis and interpretation

Imprinted DMRs are defined as regions in the genome that show differences in DNA methylation between the two alleles. At these regions one allele is completely methylated while the other remains unmethylated, which is dependent on the parent of origin. Therefore, in a normal state the expected methylation beta-value (assessed on methylation array) at imprinted DMRs, is expected to be ~ 0.5 and any deviation from this i.e., gain or loss of methylation levels indicates imprinting dysregulation.

For studying imprinting deregulation in NB, we focused on 49 of the 50 known imprinted DMRs previously reported by Mora et al. (2018) [30]. This study compared the methylation profiles of normal biparental and reciprocal uniparental diploidy samples and reported 789 CpG probes mapping to 50 known imprinted DMRs. Out of 789 CpG probes, 704 CpG positions overlapping 49 imprinted DMRs were overlapping with the NB and foetal adrenal methylation data and thus were further investigated in this study.

Herein, we name the imprinted DMRs based on the genes that are mapping to that region (Table 2). Names of all the genes are used in the nomenclature if a region was associated with more than one gene. For example, NNAT:TSS-DMR that maps to the genes *NNAT* and *BLAP* is named as *NNAT*/*BLAP*. DMRs that do not overlap any gene or overlap a pseudogene are named as the original DMR name. Similarly, when the overlapping gene is common between two or more DMRs, the original DMR name is used in the nomenclature.

**Table 2:** Genomic location and overlapping gene names and function of the imprinted differentially methylated regions (DMRs)

Imprinted DMRs	Methylated Allele	Chr	Region covered	No of CpG	Overlapping genes	Gene function	Role in cancer/other disease
PPIEL:Ex1-DMR	M	1	Chr1:40024971–40025415	4	*LOC728448*	Pseudogene	–
DIRAS3:Ex2-DMR	M	1	Chr1:68512539–68513063	8	*DIRAS3*	Tumour suppressor	Downregulated in multiple cancers [59, 60]
DIRAS3:TSS-DMR	M	1	Chr1:68515788–68517273	21	*DIRAS3*	As above	As above
GPR1-AS:TSS-DMR	M	2	Chr2:207068254–207068409	2	*GPR1*	Neuronal stem-cell proliferation and differentiation	Promotes cancer cell proliferation and invasion [61, 62]
ZDBF2/GPR1:IG-DMR	P	2	Chr2:207116070–207129158	8	–	–	–
NAP1L5:TSS-DMR	M	4	Chr4:89618533–89619236	14	*NAP1L5; HERC3*	*NAP1L5:* Promotes proliferation of neural progenitors and inhibits neuronal differentiation during cortical development *HERC3*: ligase and ubiquitin-protein transferase activity	*NAP1L5:* Potential tumour suppressor in hepatocellular carcinomas [63] *HERC3:* Potential tumour suppressor in colorectal cancer [64]
VTRNA2-1:DMR	M	5	Chr5:135414858–135416613	17	*MIR886*	Cell growth regulation	Often hypermethylated and repressed in cancer [65–67]
FAM50B:TSS-DMR	M	6	Chr6:3849095–3850106	24	*FAM50B*	Role in the circadian clock	–
PLAGL1:alt-TSS-DMR	M	6	Chr6:144328421–144329887	14	*HYMAI; PLAGL1*	*HYMAI:* non-coding *PLAGL1:* Transcriptional activator. Encodes a C2H2 zinc finger protein that functions as a suppressor of cell growth	Transient neonatal diabetes mellitus [68]
IGF2R:Int2-DMR	M	6	Chr6:160427501	1	*IGF2R*	G protein-coupled receptor activity and enzyme binding	
WDR27:Int13-DMR	M	6	Chr6:170054730–170055332	2	*WDR27*	Cell signalling	–
GRB10:alt-TSS-DMR	M	7	Chr7:50849168–50850870	8	*GRB10*	Insulin receptor signalling cascade and nervous system development	Novel oncogene in glioma [69]
PEG10:TSS-DMR	M	7	Chr7:94285642–94287242	50	*SGCE; PEG10*	*SGCE*: calcium ion binding *PEG10*: cell proliferation, differentiation, and apoptosis	*PEG10*: Overexpressed in several cancers and promotes tumor progression [70–73]
MEST:alt-TSS-DMR	M	7	Chr7:130130122–130133110	54	*MESTIT1; MEST*	*MESTIT1:* non-coding RNA involved in the regulation of *MEST* expression during development *MEST*: embryo development	*MESTIT1:* Deregulated in breast cancer and promotes cancer progression [74] MEST: esophageal squamous cell carcinoma [75]
SVOPL:alt-TSS-DMR	M	7	Chr7:138348774–138348981	2	*SVOPL*	Transmembrane transporter activity	Breast cancer [76], colorectal cancer [77]
HTR5A:TSS-DMR	M	7	Chr7:154862770–154863381	6	*HTR5A*	Functions as a neurotransmitter	Downregulated in high grade glioma [78]
ERLIN2:Int6-DMR	M	8	Chr8:37605359–37605978	7	*LOC728024; ERLIN2*	*LOC728024: *Pseudogene *ERLIN2*: cell cycle progression	*ERLIN2:* Overexpressed in aggressive breast cancer and promotes tumour growth [79]
PEG13:TSS-DMR	M	8	Chr8:141108607–141110900	7	*TRAPPC9*	NF-kappa-B signaling and neuronal cells differentiation	Promotes tumourigenesis in cancer cells [80]
FANCC:Int1-DMR	M	9	Chr9:98075492	1	*FANCC*	Maintenance of normal chromosome stability	–
INPP5F:Int2-DMR	M	10	Chr10:121578137–121578639	4	*INPP5F*	Phosphoric ester hydrolase activity	Potential tumour suppressor in glioblastoma [81]
H19/IGF2:IG-DMR	P	11	Chr11:2019079–2024126	43	*H19; MIR675*	*H19:* Long non-coding RNA *MIR675:* post-transcriptional regulation of gene expression	*H19*: Overexpressed in multiple cancers and facilitates several cellular processes including cell proliferation, migration, invasion [82–84] *MIR675:* predicted target of H19 [85]
IGF2:Ex9-DMR	M	11	Chr11:2153991–2154952	9	*INS-IGF2; IGF2*	*INS-IGF2: *AMP-activated protein kinase signaling *IGF2: *growth factor activity	*INS-IGF2: *promotes cell proliferation in lung cancer [86] *IGF2:* Overexpressed in multiple cancers and associated with poor prognosis [87–90]
IGF2:alt-TSS-DMR	M	11	Chr11:2168625	1	*IGF2AS; INS-IGF2*	*IGF2AS:* RNA gene *INS-IGF2: *As above	*IGF2AS:* potential tumour suppressor in prostate cancer [91]
KCNQ1OT1:TSS-DMR	M	11	Chr11:2720229–2722195	26	*KCNQ1; KCNQ1OT1*	*KCNQ1:* Transmembrane transporter binding *KCNQ1OT1:* long non-coding RNA that regulates transcription of multiple target genes	*KCNQ1OT1*: acts as an oncogene in colon cancer [92]
RB1:Int2-DMR	M	13	Chr13:48892551–48895478	13	*RB1*	Negative regulator of cell cycle	Tumour suppressor, deregulated in multiple cancers [39, 93–97]
MEG3:TSS-DMR	P	14	Chr14:101290556–101293856	32	*MEG3*	Interacts with the tumour suppressor p53 and regulates p53 target gene expression	Deregulated in multiple cancers [98]
MEG8:Int2-DMR	M	14	Chr14:101370989	1	*MEG8*	Long non-coding RNA	Involved in cancer progression in multiple cancers [99]
MKRN3:TSS-DMR	M	15	Chr15:23807180–23812334	11	*MKRN3*	Ligase activity	Tumour suppressor frequently mutated in non–small cell lung cancer [100]
MAGEL2:TSS-DMR	M	15	Chr15:23892574–23893742	6	*MAGEL2*	Ubiquitin-protein transferase activity	Downregulated and associated with poor prognosis in glioma [101]
NDN:TSS-DMR	M	15	Chr15:23931451–23932758	8	*NDN*	Growth suppressor, also interacts with p53 and works in an additive manner to inhibit cell growth	Tumour suppressor [41, 102]
SNRPN:alt-TSS-DMR	M	15	Chr15:25068738–25069376	7	*SNRPN*	pre-mRNA processing and tissue-specific alternative splicing	Promotes tumour proliferation and migration in colorectal cancer [103]
SNRPN:Int1-DMR1	M	15	Chr15:25,093,244–25093521	4	*SNRPN*	As above	As above
SNRPN:Int1-DMR2	M	15	Chr15:25123287–25123688	4	*SNRPN*	As above	As above
SNURF:TSS-DMR	M	15	Chr15:25200253–25201732	6	*SNRPN, SNURF*	*SNURF*: Processing of Capped Intron-Containing Pre-mRNA	*SNURF*: Breast cancer [104]
IGF1R:Int2-DMR	M	15	Chr15:99408636–99409506	7	*IGF1R*	Tyrosine kinase activity	Cancer promoting in lung cancer [105]
ZNF597:3' DMR	M	16	Chr16:3481970–3482078	2	*–*	–	–
ZNF597:TSS-DMR	P	16	Chr16:3493133–3494155	12	*ZNF597; NAT15*	*ZNF597:* nucleic acid binding *NAT15: *acyltransferase activity	–
ZNF331:alt-TSS-DMR1	M	19	Chr19:54040774–54042165	12	*ZNF331*	Nucleic acid binding	Novel tumour suppressor in gastric cancer and potential biomarker in colorectal cancer detection [106, 107]
ZNF331:alt-TSS-DMR2	M	19	Chr19:54057208–54058085	4	*ZNF331*	As above	As above
PEG3:TSS-DMR	M	19	Chr19:57349204–57352785	34	*ZIM2; PEG3; MIMT1*	*ZIM2, PEG3: *transcriptional regulation	–
MCTS2P:TSS-DMR	M	20	Chr20:30134929–30135362	8	*HM13; PSIMCT-1*	*HM13:* Protein homodimerization activity and peptidase activity *PSIMCT-1:* Pseudogene	*HM13:* Potential predictive biomarker in hepatocellular carcinoma [108]
NNAT:TSS-DMR	M	20	Chr20:36148604–36150061	36	*BLCAP; NNAT*	*BLCAP:* reduces cell growth by stimulating apoptosis *NNAT*: Maturation or maintenance of the overall structure of the nervous system	*BLCAP*: acts as tumour suppressor gene in many cancers [109, 110] *NNAT*: altered in several cancers including non-small cell lung cancer [111], glioblastoma [112], medulloblastoma [113], neuroblastoma [38]
L3MBTL1:alt-TSS-DMR	M	20	Chr20:42142417–42143502	26	*L3MBTL*	Function as methyl-lysine readers, associated with the repression of gene expression	Candidate tumour suppressor gene myeloid cancers [114]
GNAS-NESP:TSS-DMR	P	20	Chr20:57414039–57418015	21	*GNASAS; GNAS*	*GNASAS:* long non-coding RNA *GNAS-*GTP binding and obsolete signal transducer activity	GNAS: Promotes cancer progression in hepatocellular carcinoma [115]
GNAS-AS1:TSS-DMR	M	20	Chr20:57425979–57427973	60	*GNASAS; GNAS*	As above	As above
GNAS-XL:Ex1-DMR	M	20	Chr20:57429858–57431303	5	*GNAS*	As above	
GNAS A/B:TSS-DMR	M	20	Chr20:57463265–57465175	40	*GNAS*	As above	As above
WRB:alt-TSS-DMR	M	21	Chr21:40757691–40758208	4	*WRB*	Peroxisomal lipid metabolism	–
SNU13:alt-TSS-DMR	M	22	Chr22:42077939–42078723	8	*NHP2L1*	Pre-mRNA splicing	Promotes cell proliferation in triple negative breast cancer [116]

*DMRs* differentially methylated regions, M: maternal, P: paternal, chr: chromosome

To identify the regions with aberrant methylation pattern in NB, a probe-wise investigation was performed across the imprinted DMR. At a probe, NB cases were considered to have undergone gain of methylation (GOM) or loss of methylation (LOM) if the probe methylation level was above or below 0.8/0.2 and were classified as “GOM” and “LOM”, respectively. Moderate methylation events were also calculated by comparing beta-values of NB tumours to the probe mean of control samples. NB cases with probe methylation level below the healthy controls’ mean 2 standard deviation (SD) CI were classified as “Intermediate LOM” and those above 2 SDs CI were classified as “Intermediate GOM” [31]. Cases that did not meet the above criteria were grouped as “No change”, suggesting a normal methylation pattern. Since the imprinted DMRs were associated with more than one CpG probe, to get a region-wise indication of methylation alteration, the NB samples were further grouped based on the event that was supported by more than 60% of probes in that region. We defined the most altered regions as the regions that displayed alteration, either GOM or LOM in > 30% of NB samples in both discovery and the validation set.

### Allele-specific copy number alteration profiles

The allele-specific CNA profiles were derived using ASCAT [32]. Briefly, this algorithm takes total signal intensity (Log R) and allelic contrast represented by B allele frequency (BAF) from tumour and matched germline as input. As the first step, the germline data was used to determine the germline homozygous SNP array probes. The data was then segmented using ASPCF segmentation algorithm and an “ASCAT profile” was calculated across all assayed loci. The output ASCAT profile was reported as “*nMajor*”, “*nMinor*” and “*ploidy*” that referred to the number of major alleles, number of minor alleles and the total copy number in specific regions, respectively, which was the key output for our analysis and allowed for an accurate derivation of gains, losses, copy-number-neutral events, and loss of heterozygosity for all the samples.

### Predicted methylation analysis

CNAs that overlapped with the imprinted DMRs were investigated in this study. Prediction of methylation levels based on allele-specific CNAs was done as described [31] and was based on the following assumptions (a) if no epimutations have occurred, then methylation beta-values will be completely guided by the copy number and parental origin, in a normal diploid cell (2n), methylation will be ~ 50% (one methylated and one unmethylated allele) whereas in a cell with aneuploidy, for example 4n, the beta-values will be in a ratio of 75:25 or 25:75%, depending on the parental origin of the minor allele and (b) in all cases of copy number neutral loss of heterozygosity, beta-values will be close to zero or 1 irrespective of the total chromosome number.

Firstly, “minor methylation value” were calculated as the ratio of minor allele count and total allele count for each sample across each region. We then checked the HM450K methylation beta-value for the corresponding region. For each region, if the HM450K methylation beta-value for majority of the probes was below 0.5 or equal to 0.5, minor allele was assumed to be the methylated allele and the CNA-based predicted methylation value was reported as equal to the minor methylation value (ratio of minor allele count and total allele count). If the corresponding HM450K value was above 0.5, major allele was assumed to be the methylated allele and the CNA-based predicted methylation value was reported as 1-minor methylation value. Next, to know what proportion of methylation alterations can be explained by the underlying CNAs, a linear regression analysis was performed with observed array-based (HM450K) methylation value as outcome and CNA-based predicted methylation as predictor. A coefficient of determination (adjusted R^2^) was obtained for each region based on the linear regression analysis that explain the degree to which the predictor variable (CNA-based predicted methylation) explains the variation of the output variable (array-based methylation value). The value of adjusted R^2^ ranges from 0 to 1, where 0 indicates that the outcome cannot be explained by the predictor variable and 1 indicates vice versa. A negative adjusted R^2^ suggests that the model is a poor fit to the data.

### Gene expression analysis

Kaplan–Meier analysis with calculation of log rank test for overall survival probability for NB in relation to the expression levels of genes, which were overlapping the seven DMRs that showed significant association with overall survival in this study were performed using the Kaplan scan cutoff method in ‘R2: Genomic Analysis and Visualization Platform’ (http://r2.amc.nl). The prognostic significance on overall survival of the DMRs were investigated using the three publicly available datasets; “Tumor Neuroblastoma SEQC-498 custom—GSE49710 (n = 498), “Tumor Neuroblastoma Maris 101 custom”—GSE3960 (n = 101) and, “Tumor Neuroblastoma Kocak 649 custom—GSE45547 (n = 649). The Kaplan scan cutoff method examines every increasing expression value as cutoff for log rank test in order to find the optimal segregation point of two groups based on gene expression. This method then presents the most statistically significant cutoff with corresponding Bonferroni corrected P-value together with the initial non-corrected P-value.

### Survival analysis

Survival information was available for all 185 patients from the TARGET dataset and 68/85 (80%) samples from the validation cohort. Follow-up started at the date of diagnosis and ended at the date of death or end of follow-up, whichever came first. Methylation alterations at all 49 imprinted regions and its role in disease prognosis were tested. We compared the overall survival probability of the imprinting methylation groups in samples from the TARGET dataset with matching survival information using log-rank test. Survival analyses were undertaken using the R package Survival [33]. P-value < 0.05 was considered significant. Cox proportional hazards regression models were used to calculate hazard ratios (HRs) and 95% confidence intervals (CI) for the association between methylation alterations at the imprinted DMRs and risk of death. Multivariate model with adjustment for *MYCN* amplification, 11q deletion and age at diagnosis were fitted.

### Statistical analysis

Pearson’s Chi-squared tests were performed to assess the association between methylation changes at the imprinted regions that were most altered (i.e. underwent LOM or GOM in > 30% of samples) and clinical and molecular features of the patients/tumours. The P-values were adjusted for multiple hypotheses testing using Benjamini-Hochberg (BH) method. P-value < 0.05 was considered significant.

## Results

### Study participants

The samples included in this study were obtained from the TARGET (n = 185) and German Neuroblastoma Trial (n = 99), merged as discovery set and a local cohort of NB tumours (n = 85), used as validation set.

### Imprinting aberrations in neuroblastoma

To study imprinting deregulation in NB, 704 CpG positions overlapping 49 imprinted DMRs as previously reported by Mora et al. (2018) [30] were investigated in the discovery set (n = 284). The details of the imprinted regions, genomic location, overlapping genes and their function is presented in Table 2. Imprinted DMRs were present in all chromosomes except chromosome 3, 12, 17 and 18. Most of the DMRs were associated with at least 2 CpG sites. Out of 704 CpG sites, 672 (95%) sites mapped to a gene (Table 2). The CpG sites were mainly located in the promoter associated regions including transcription start site 1500 (TSS1500) (37%), 5’ untranslated region (5’UTR) (21%), TSS200 (13%), 3’UTR (10%) and 1st exon (8%). Twelve percent of the CpG sites were in the gene body region.

To identify regions with altered methylation patterns, a probe-wise investigation was performed, and the samples were assigned to groups; gain of methylation (GOM) and loss of methylation (LOM), when the probe-wise methylation level was above 0.8 and below 0.2, respectively. We also defined groups with moderate changes in the methylation, namely “Intermediate GOM” and “Intermediate LOM”, based on if the methylation level of a NB sample was above or below 2 SD of the mean of the healthy control’s (healthy foetal adrenal tissue). Samples that did not meet any of the above criteria were grouped as “No change”, i.e. no methylation aberrations were observed.

Imprinted DMRs are methylated at either the maternal or paternal allele and therefore average methylation in these regions in a normal state is expected to be ~ 0.5. In all DMRs, the average methylation beta-value in the control samples (n = 11) ranged, as expected, between 0.4 and 0.6 except for four regions (*GPR1*, *MKRN3*, NNAT/*BLCAP* and IGF2:alt-TSS-DMR) where the average methylation of control adrenal samples was higher than 0.7. Similarly, five regions had methylation values of < 0.4 in control adrenal samples (IGF2:Ex9-DMR, *IGF2R*, *MIR886*, SNRPN:Int1-DMR1 and *SVOPL*) (Supplementary Table 1). However, since imprinting patterns are tissue specific, selecting the most suitable control for such analysis is challenging. Considering that NB originates from neural crest cells, foetal adrenal gland tissue may not be a suitable control for this analysis, and therefore we chose not to remove regions or disregard the results based on the methylation value of the adrenal control tissue. In our analysis strategy, we used the control group to define the moderate methylation changes, while the more stronger methylation changes defined as GOM and LOM are based on the probe-wise absolute beta-value of the NB samples. The most altered regions are also reported based on the stronger methylation changes. A heatmap displaying the beta-value of NB samples in the discovery set (n = 284), control adrenal samples (n = 11) and mUPD (n = 1) and pUPD (n = 2) samples are presented in Supplementary Fig. 1.

**Fig. 1:**
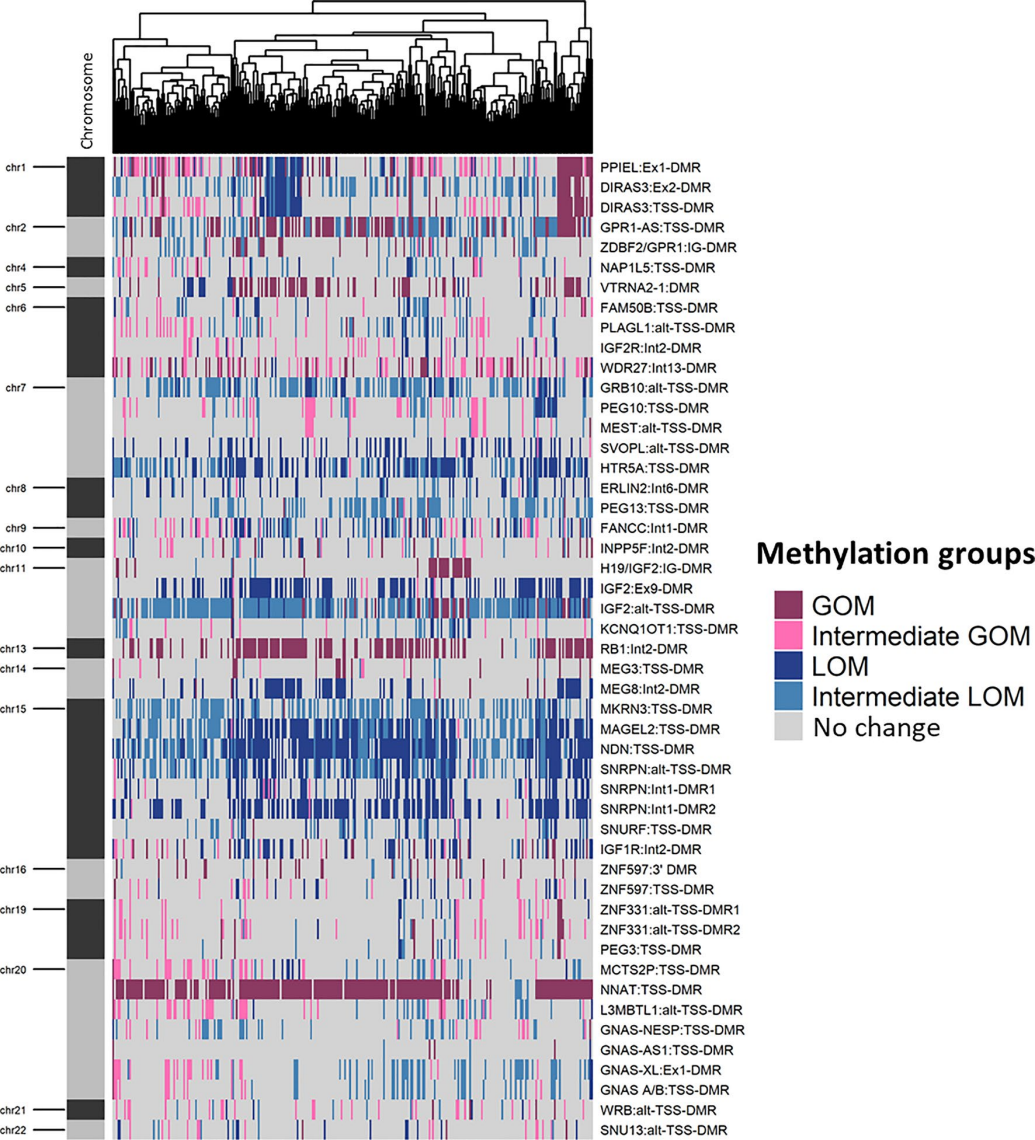
Methylation pattern of Neuroblastoma (NB) tumour samples. Heatmap shows the methylation patterns of neuroblastoma (NB) tumour samples in the discovery set (n = 284) that included samples from Therapeutically Applicable Research to Generate Effective Treatments (TARGET) and German Neuroblastoma Trial, across 49 imprinted differentially methylated regions (DMRs). Annotation of Imprinted DMRs by genomic position and location in the context of chromosome are marked on the heatmap. Methylation changes, namely gain of methylation (GOM), loss of methylation (LOM), Intermediate GOM, Intermediate LOM and No change identified in the analysis are shown in the heatmap as indicated in the colour key

At least one of the NB samples displayed methylation changes in any of the 49 DMRs and no region was unaffected (Fig. 1). LOM was more commonly observed in NB samples than GOM. The top five regions where LOM was frequently observed were *NDN* (50% LOM, 26% Intermediate LOM), SNRPN:Int1-DMR2 (50% LOM) and *MAGEL2* (37% LOM, 28% Intermediate LOM) located on chromosome 15, IGF2:Ex9-DMR (41% LOM) located at chromosome 11p, and *HTR5A* (34% LOM, 21% Intermediate LOM) located at chromosome 7q (Supplementary Table 1). Methylation alterations in these regions were unidirectional i.e. no NB sample underwent GOM at any of these sites, and there were only four cases of Intermediate GOM. Interestingly, six of these top ten regions with LOM events were located on chromosome 15.

As stated, GOM was less commonly observed in the NB samples but did occur. The region with the highest frequency (75%) of GOM was *NNAT/BLCAP* located at chromosome 20q. Other regions with GOM were *RB1* (46%) located on 13q, *GPR1* (33%) located on 2q, *MIR886* (25%) located on 5q, PPIEL:Ex1-DMR (18% GOM, 17% Intermediate GOM) located on 1p and *WDR27* (15% GOM, 18% Intermediate GOM) located on 6q (Fig. 1). In contrast, there were some regions that were largely unaffected by any methylation events. These included GNAS-AS1:TSS-DMR, *ZIM2/PEG3/MIMT1*, *H19/MIR675*, *MEG3* and ZNF331:alt-TSS-DMR1, where more than 90% of the NB samples had unaltered methylation levels.

### Replication in local cohort

**Fig. 2:**
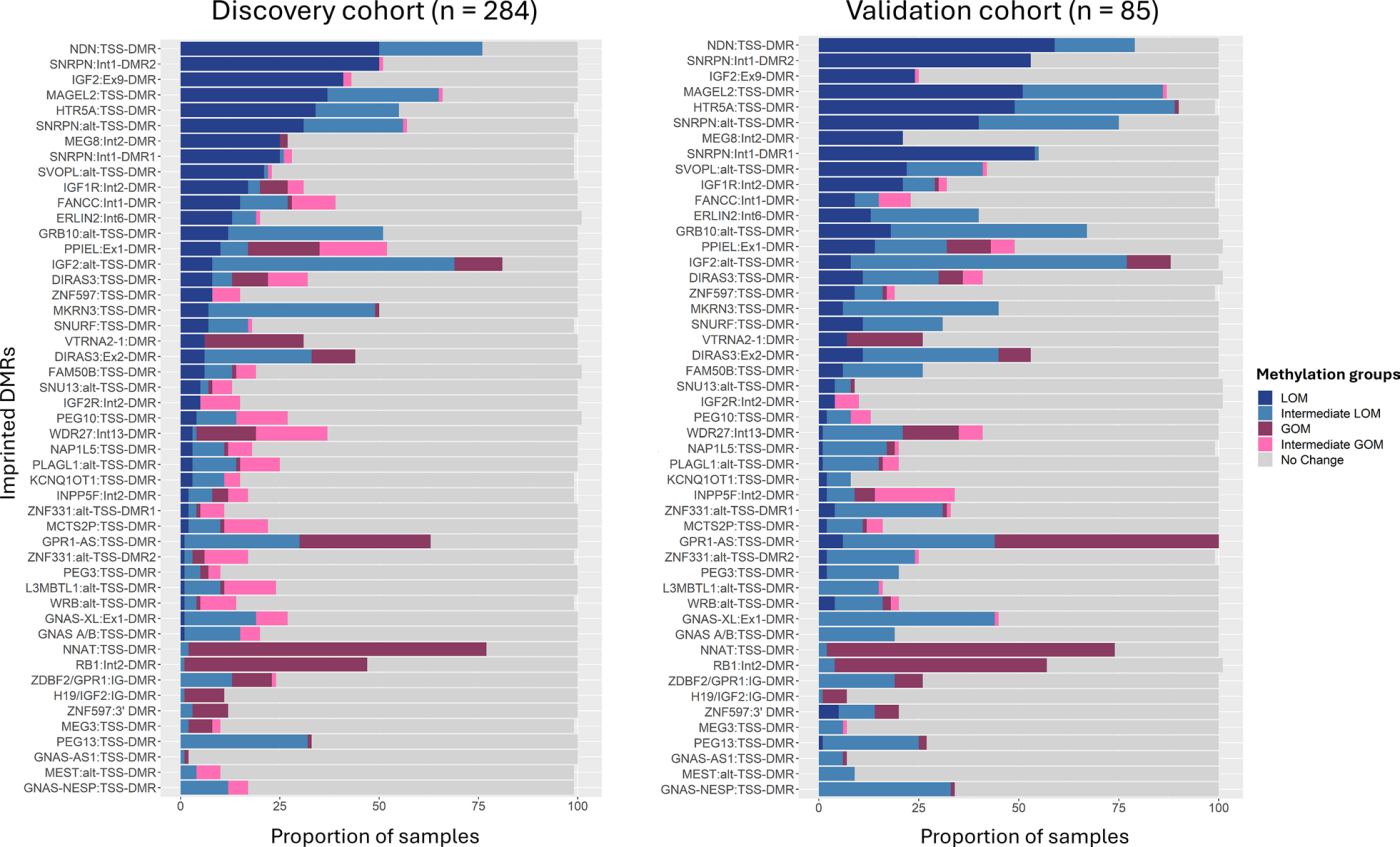
Methylation alterations in the validation set. Stacked barplots present a comparison of the methylation patterns of neuroblastoma (NB) samples in the discovery set (n = 284) and the validation set (n = 85). Methylation changes displayed by the NB tumours that included gain of methylation (GOM), loss of methylation (LOM), Intermediate GOM, Intermediate LOM or No change at the imprinted differentially methylated regions (DMRs) are presented as indicated in the colour key. The imprinted regions are ordered by proportion of samples displaying LOM and GOM in the public dataset with the regions displaying the most alterations in the public dataset shown on the top

The results from the methylation analysis were validated in a local cohort of 85 NB samples. Methylation profiles of NB samples at the imprinted DMRs had a similar pattern in the validation set (Fig. 2). Consistent with the discovery set, LOM was prominently observed at DMRs on chromosome 15. Regions where LOM was observed frequently (> 30% of the NB samples) in the discovery set also ranked highly in the validation set. Regions with frequent GOM events (> 30% of the NB samples) remained consistent in the validation set (Fig. 2). Methylation alterations at the imprinted DMRs in NB tumours from the three datasets; TARGET, German Neuroblastoma Trial, and the local cohort is presented in individual heatmaps (Supplementary Fig. 2A–C) and sample proportions in the individual datasets in Supplementary Table 2.

### Association with tumour features and established molecular groups

After identifying the imprinted DMRs with the most altered methylation, we next investigated whether the identified methylation changes in these regions were associated with clinical or genomic features of the tumours. Pearson’s Chi-squared tests were performed to assess the association between methylation changes at the imprinted regions that were most altered (i.e. underwent LOM or GOM in > 30% of samples) and tumour characteristics including age group at diagnosis, tumour stage, 11q deletion or *MYCN* amplification status. Association with methylation-based subclasses as defined by the molecular neuropathology classifier were also assessed [34]. Since the discovery set and the validation set displayed largely similar methylation pattern at the imprinted regions, the two sets were combined to increase the sample size and the power of the correlation tests.

**Fig. 3:**
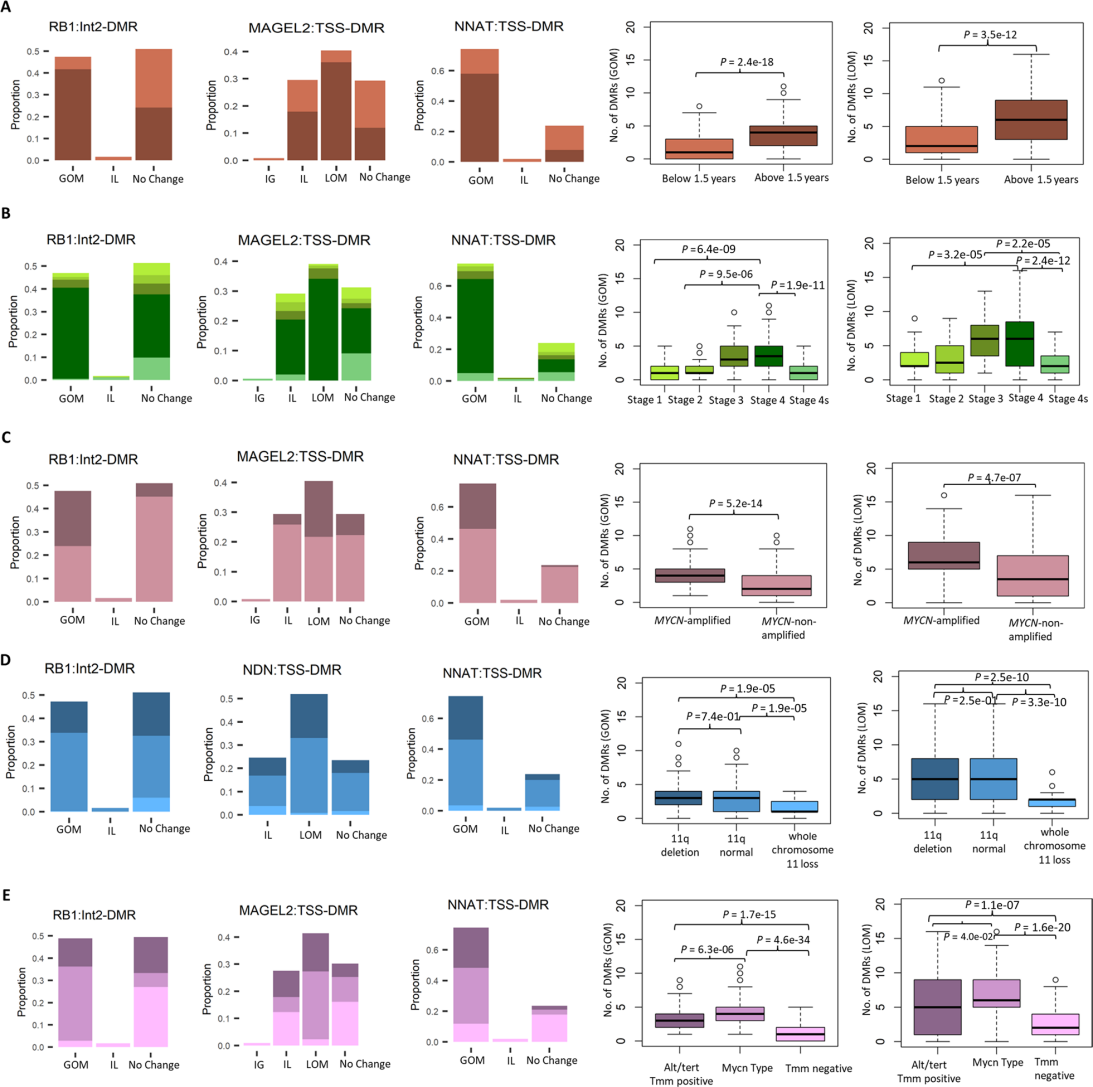
Methylation alterations at the imprinted regions and association with clinical and genomic features. Bar plots show the frequency of 369 NB samples in a combined cohort of the discovery set and the validation set grouped based on **A** age at diagnosis **B** tumour stage **C** *MYCN* amplification **D** 11q deletion and **E** Molecular subclasses (defined based on the molecular neuropathology classifier), stratified by methylation alterations at the imprinted differentially methylated regions (DMRs) namely, gain of methylation (GOM), loss of methylation (LOM), Intermediate LOM (IL), Intermediate GOM (IG) and No change. Box plots on the right panel show the difference in the total number of imprinted DMRs undergoing methylation changes (GOM or LOM) between the NB tumours in different clinical groups mentioned above. P-values were corrected for multiple hypotheses testing using Benjamini–Hochberg method

Significant associations between methylation alterations at the imprinted DMRs and clinical and genomic features were observed (Supplementary Table 3). Change in methylation levels for example at *RB1* (BH adjusted *P* = 8.5e-17), *NNAT/BLCAP* (BH adjusted *P* = 3.5e-16) and *MAGEL2* (BH adjusted *P* = 2.0e-15) correlated strongly with higher age at diagnosis. Tumours that underwent methylation changes at these regions were diagnosed at an older age (> 1.5 years) compared to the tumours that had unaltered methylation patterns at these sites (Fig. 3A). A significant difference in the total number of imprinted DMRs that underwent methylation changes was also observed between group of patients with age at diagnosis above and below 1.5 years. Patients with older age at diagnosis (above 1.5 years) had significantly larger number of regions that underwent LOM (*P* = 3.5e-12) and GOM (*P* = 2.4e-18) when compared with patients that were below 1.5 years at diagnosis (Fig. 3A). Methylation changes in these regions also correlated with stage 4 tumours. For cases that displayed GOM at *RB1* and *NNAT/BLCAP*, 85% and 80%, respectively were classified as stage 4 tumours, although we also see stage 4 tumour cases (41%) in the “No change” group at *RB1*. Loss of methylation at *MAGEL2* was predominantly associated with stage 4 tumours (87% of cases in LOM group and 63% of cases in the Intermediate LOM group) (Fig. 3B). Comparing the total number of imprinted DMRs undergoing methylation changes between the different tumour stages, we found that in comparison with stage 1 tumours, stage 4 and stage 3 tumour cases had significantly larger number of imprinted DMRs undergoing LOM (stage 4 vs stage 1, BH adjusted *P* = 3.2e-05, stage 3 vs stage 1, BH adjusted *P* = 0.0001) and GOM (stage 4 vs stage 1, BH adjusted *P* = 6.4e-09, stage 3 vs stage 1, BH adjusted *P* = 1.3e-04) (Fig. 3B). On the other hand, methylation changes at imprinted regions were relatively less frequent in stage 4 s tumours. In comparison with stage 4 and stage 3 tumours, stage 4 s tumour cases had significantly fewer number of imprinted DMRs undergoing LOM (stage 4 vs stage 4 s, BH adjusted *P* = 2.4e-12 and stage 3 vs stage 4 s, BH adjusted *P* = 2.2e-05) and GOM (stage 4 vs stage 4 s, BH adjusted *P* = 1.9e-11 and stage 3 vs stage 4 s, BH adjusted *P* = 3.0e-04) (Fig. 3B).

Methylation alterations at *RB1*, *NNAT/BLCAP* and *MAGEL2* also correlated significantly with amplification of *MYCN*. Of the cases that displayed GOM at *RB1* and *NNAT/BLCAP*, 50% and 38%, respectively, had amplification of *MYCN*, whereas of the cases with no change in methylation at these regions only 11% and 5%, respectively, had amplification of *MYCN*. At *MAGEL2*, 46% of cases with LOM and 12% of cases with Intermediate LOM had amplification of *MYCN* (Fig. 3C). *MYCN*-amplified NB cases also had significantly larger number of DMRs that displayed LOM (*P* = 4.7e-07) and GOM (*P* = 5.e-14) compared to the cases with no amplification of *MYCN* (Fig. 3C). In case of 11q deletion, methylation changes at *NDN* and *NNAT/BLCAP* correlated strongly with 11q deletion. Of the cases that displayed LOM at *NDN* and GOM at *NNAT/BLCAP*, 31% and 38%, respectively, had deletion of 11q, whereas of the cases with no change in methylation at these regions 23% and 16%, respectively, had deletion of 11q. At *RB1* on the other hand, the majority of cases that displayed GOM had normal 11q (71%) (Fig. 3D). In terms of total number of imprinted DMRs that underwent methylation changes, no significant difference was observed between the cases that had 11q deletion and the cases that had normal copy of the 11q chromosome. However, tumour cases that had a loss of the whole chromosome 11 harboured significantly lower number of imprinted DMRs undergoing methylation changes in comparison to both 11q-deleted (LOM: BH adjusted *P* = 2.5e-10, GOM, BH adjusted *P* = 1.9e-05) and 11q-normal (LOM: BH adjusted *P* = 3.3e-10, GOM, BH adjusted *P* = 1.9e-05) cases (Fig. 3D).

DNA methylation-based classification is successfully used for central nervous system tumour classification [34–36]. In the current Molecular Neuropathology (MNP) classifier, NB can be classified into three methylation subclasses namely *Alt/Tert Tmm Positive, Mycn Type* and *Tmm Negative*. *Atl/Tert Tmm Positive* molecular subclass includes the NB tumours that show an activation of the telomerase maintenance mechanism (Tmm) and is associated with a poor outcome. On the other hand, the *Tmm Negative* NB tumours lack this mechanism and thus have better prognosis [37]. The *Mycn Type,* as the name suggests, includes tumours that harbor amplification of the *MYCN* gene. In the combined dataset (discovery and validation), 37% of NB tumours were classified as *Mycn Type*, 27% as *Alt/Tert Tmm Positive* and 30% as *Tmm Negative.* There were 21 cases that remained unclassified due to subclassification score < 0.5 and were subsequently excluded from the assessment in the correlation between methylation-based subclasses and the imprinting groups at the imprinted DMRs.

Methylation alterations (GOM or LOM) at *NNAT/BLCAP* (BH adjusted *P* = 2.0e-08), *RB1* (BH adjusted *P* = 2.4e-14) and *MAGEL2* (BH adjusted *P* = 5.3e-08) showed an enrichment for the methylation subclass *Mycn Type.* Interestingly, most of the tumours that retained their methylation profile (No change group) at these DMRs were classified as *Tmm negative* (76% of cases with no change at *NNAT/BLCAP*, 55% at *RB* and 53% at *MAGEL2*) (Fig. 3E). The tumours, however, that harboured methylation alterations at these regions were mainly classified as *Mycn Type.* At *RB1*, 68% cases that underwent GOM were classified as *Mycn Type,* 26% as *Alt/Tert Tmm Positive* and 6% as *Tmm Negative.* Similarly at *NNAT/BLCAP*, 49% cases that underwent GOM were classified as *Mycn Type,* 35% as *Alt/Tert Tmm Positive* and 16% as *Tmm Negative.* At *MAGEL2*, 60% cases with LOM were classified as *Mycn Type,* 34% as *Alt/Tert Tmm Positive* and 5% as *Tmm Negative.* The difference was also evident when we compared the total number of DMRs that underwent methylation changes between the molecular subclasses. *Tmm negative* had significantly fewer DMRs that were affected by methylation changes (LOM or GOM) when compared with *Alt/Tert Tmm Positive* (LOM, BH adjusted *P* = 1.1e-07 and GOM, BH adjusted *P* = 2.5e-15) and *Mycn Type* (LOM, BH adjusted *P* = 1.6e-20 and GOM, BH adjusted *P* = 1.4e-33) subclasses (Fig. 3E). The *MYCN Type* group had the largest number of imprinted regions affected by methylation changes among the molecular subclasses (Fig. 3E).

### Methylation changes at imprinted DMRs and association with overall survival

**Fig. 4:**
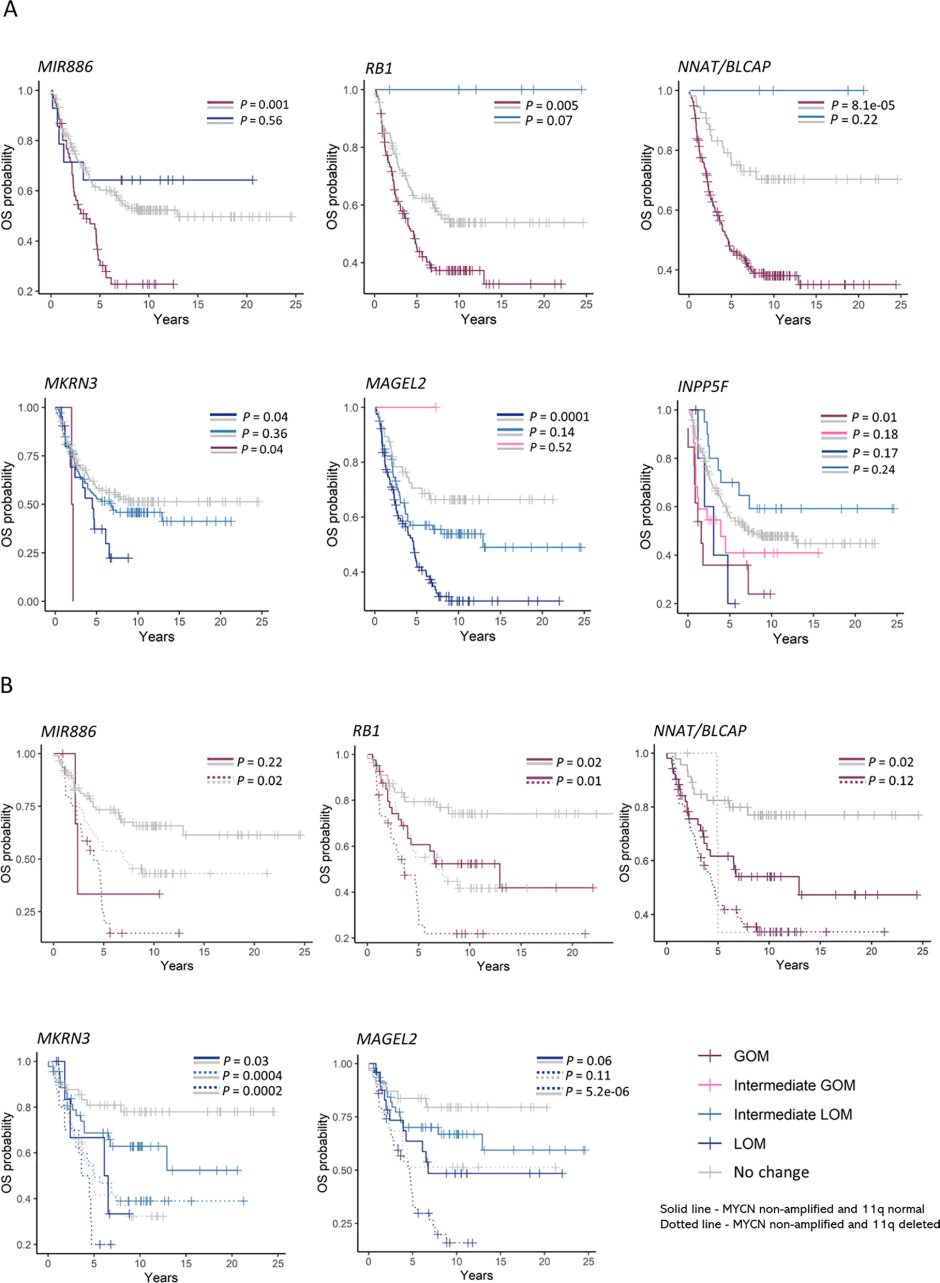
Overall survival probability. **A** Kaplan–Meier plots show the differences in overall survival probability of 253 neuroblastoma (NB) patients in the pooled cohort of samples in the Therapeutically Applicable Research to Generate Effective Treatments (TARGET) dataset and local cohort, stratified by methylation alterations at the imprinted differentially methylated regions (DMRs), which are gain of methylation (GOM), loss of methylation (LOM), Intermediate LOM, Intermediate GOM and No change. **B** Kaplan–Meier plots show the differences in overall survival probability of *MYCN* non-amplified NB patients stratified by methylation alterations at the imprinted DMRs and 11q deletion. Dotted line in the plots represents *MYCN* non-amplified NB tumours with a deletion of 11q while the solid line represents tumours that were *MYCN* non-amplified with a normal copy of 11q

To compare risk prediction of the DMRs we combined the survival data and the imprinting groups from the public (TARGET) and the local cohort. Survival data was not available for the German Neuroblastoma study. The pooled analysis indicated that methylation alteration was associated with overall survival for six DMRs*.* A loss in methylation at *MAGEL2* (*P* = 0.0001) and *MKRN3* (*P* = 0.04) was significantly associated with poorer overall survival. Methylation gains were associated significantly with shorter overall survival for *MIR886* (*P* = 0.0001), *RB1* (*P* = 0.005), *NNAT/BLCAP* (*P* = 8.1e-05) and *INPP5F* (*P* = 0.01) (Fig. 4A). Kaplan–Meier plots showing the risk prediction of DMRs in TARGET and the local cohort separately is presented in the Supplementary Fig. 3A and 3B, respectively.

To further examine the prognostic significance of the imprinted DMRs in low-risk NB group, i.e. the cases that were *MYCN* non-amplified and 11q-normal (no amplification of *MYCN* and no deletion of 11q), we compared their survival probabilities and found that the imprinted DMRs were able to further stratify *MYCN* non-amplified and 11q-normal NB cases. *MYCN* non-amplified and 11q-normal cases that underwent GOM had a shorter overall survival rate compared to the *MYCN* non-amplified and 11q-normal cases with no methylation alterations at *RB1* (*P* = 0.02) and *NNAT/BLACP* (*P* = 0.02) (Fig. 4B)*.* Similarly, at *MKRN3, MYCN* non-amplified and 11q-normal that underwent LOM had shorter survival probability compared to *the MYCN* non-amplified and 11q-normal cases that did not have any methylation changes at this site (*P* = 0.03). Although the difference was not statistically significant at *MAGEL2* and *MIR886*, we see a similar trend with *MYCN* non-amplified and 11q-normal cases undergoing LOM or Intermediate LOM having shorter overall survival probability (*P* = 0.06). At all these DMRs, the cases that had 11q-deletion and that also underwent methylation changes had the poorest survival rate (Fig. 4B).

*MYCN* amplification, 11q deletion and age at diagnosis are established prognostic biomarkers used for risk stratification in NB. To determine the contribution of the imprinted DMRs, we performed a multivariate analysis using cox proportional hazards regression model and compared the risk prediction of imprinted DMRs (*MIR886*, *RB1*, *NNAT/BLCAP*, *MAGEL2*, *MKRN3*, and *INPP5F*) after adjusting for *MYCN* amplification, 11q deletion and age at diagnosis. *MYCN* amplification status was not known for one case in the local cohort and 11q deletion status was not known for two cases in TARGET and were removed from further analysis.

**Fig. 5:**
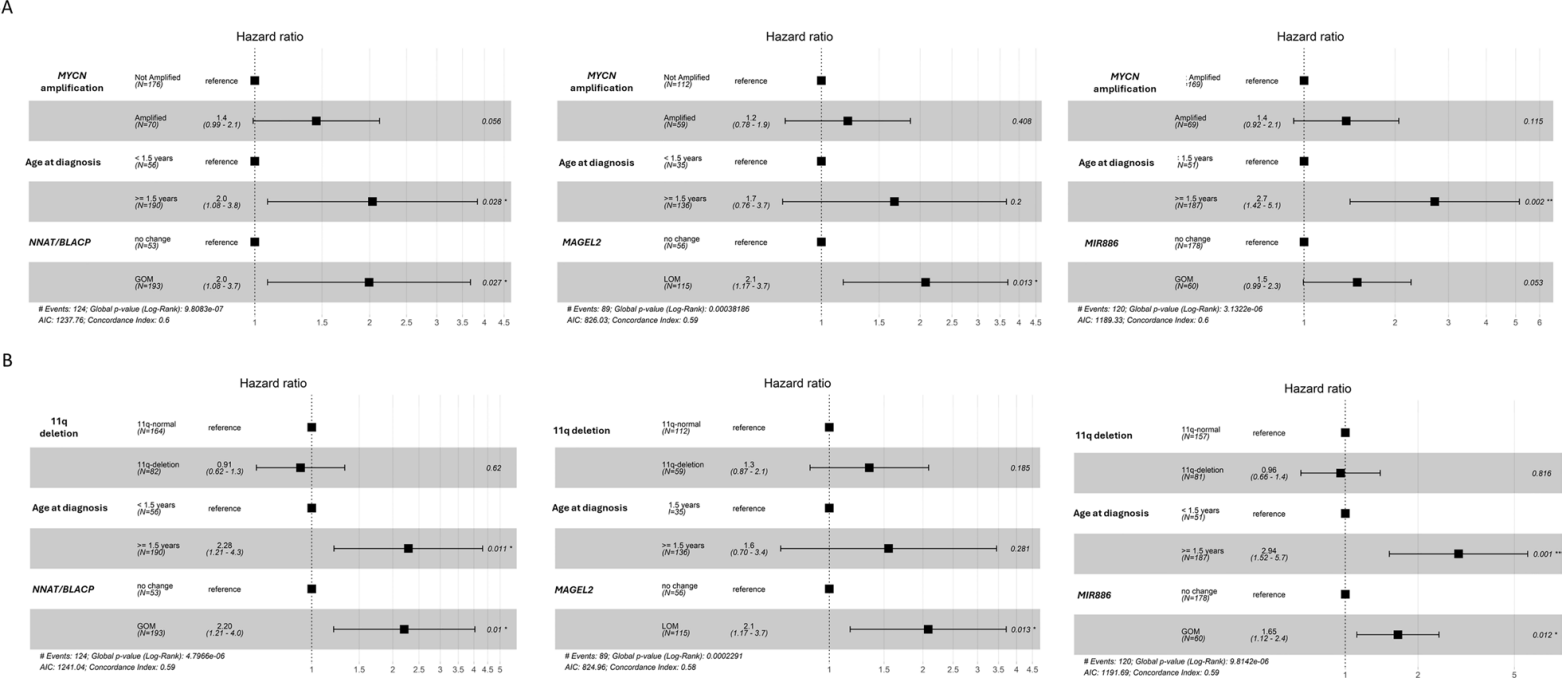
Multivariate survival analysis. Cox proportional hazards regression model of overall survival of samples in the pooled cohort (Therapeutically Applicable Research to Generate Effective Treatments (TARGET) dataset and local cohort) comparing methylation alterations at *NNAT/BLCAP*, *MAGEL2* and *MIR886* and increase in hazard after adjusting for **A** age at diagnosis and *MYCN* amplification and **B** age at diagnosis and 11q deletion

Table 3 presents the hazard ratios (HRs) of the imprinted DMRs and the established risk factors (age at diagnosis, *MYCN* amplification and 11q deletion) as a comparison. The HRs for all the six DMRs were positive indicating an increase in hazard with methylation changes at these regions. Strongest evidence of association was observed for *NNAT/BLCAP* [GOM vs No change, HR (95% CI) = 3.0 (1.7–5.3), *P* < 0.001], *MAGEL2* [LOM vs No change HR (95% CI) = 2.6 (1.5–4.4), *P* < 0.001], *RB1* [GOM vs No change, HR (95% CI) = 1.7 (1.2–2.4), *P* = 0.005] and *MIR886* [GOM vs No change, HR (95% CI) = 2.1 (1.4–3.0), *P* < 0.001]*.* After adjusting for age at diagnosis and *MYCN* amplification, the association remained consistent for *NNAT/BLCAP* [GOM vs No change, HR (95% CI) = 2.0 (1.1–3.7), *P* = 0.03] and *MAGEL2* [LOM vs No change HR (95% CI) = 2.1 (1.2–3.7), P = 0.01]. Weak evidence of association was also observed for *MIR886* [GOM vs No change, HR (95% CI) = 1.5 (1.0–2.3), *P* = 0.05] (Fig. 5A). After adjusting for age at diagnosis and 11q deletion, methylation alterations at *NNAT/BLCAP* [GOM vs No change, HR (95% CI) = 2.2 (1.2–4.0), *P* = 0.01], *MAGEL2* [LOM vs No change, HR (95% CI) = 2.1 (1.2–3.7), *P* = 0.01] and *MIR886* [GOM vs No change, HR (95% CI) = 1.7 (1.1–2.4), *P* = 0.01] were found to be associated with an increase in hazard (Fig. 5B)*.*

**Table 3:** Hazard ratios (HRs) in the pooled cohort of samples from Therapeutically Applicable Research to Generate Effective Treatments (TARGET) and the local cohort

Risk of death	HR	Lower 95%	Upper 95%	*P-value*
Age at diagnosis: Above 1.5 years (n = 190)	3.4	1.9	6.1	< 0.001
*MYCN* amplification: *MYCN* amplified (n = 70)	2.0	1.4	2.9	< 0.001
11q deletion: 11q deleted (n = 82)	1.4	1.0	2.0	0.06
*MIR886:* GOM (n = 60)	2.1	1.4	3.0	< 0.001
*RB1:* GOM (n = 132)	1.7	1.2	2.4	0.005
*NNAT/BLCAP:* GOM (n = 193)	3.0	1.7	5.3	< 0.001
*MAGEL2:* LOM (n = 115)	2.6	1.5	4.3	< 0.001
*MKRN3:* LOM (n = 21)	1.9	1.0	3.4	0.04
*INPP5F:* GOM (n = 13)	2.4	1.2	4.7	0.01

*GOM* gain of methylation, *LOM* loss of methylation

### Expression of the identified imprinted genes separates patients into risk groups

To check if the imprinted DMRs can stratify patients into risk groups based on data from gene expression arrays, we looked at the genes mapping to the identified DMRs in three datasets (Maris n = 101, Kocak n = 649 and SEQC n = 498) with gene expression data in the R2 database (https://hgserver1.amc.nl/cgi-bin/r2/main.cgi?species=hs). The prognostic significance of the genes mapping to all six DMRs identified above were tested, including *NNAT*, *RB1*, *MAGEL2*, *MKRN3* and *INPP5F.* Gene expression data was not available for *MIR886*.

**Fig. 6:**
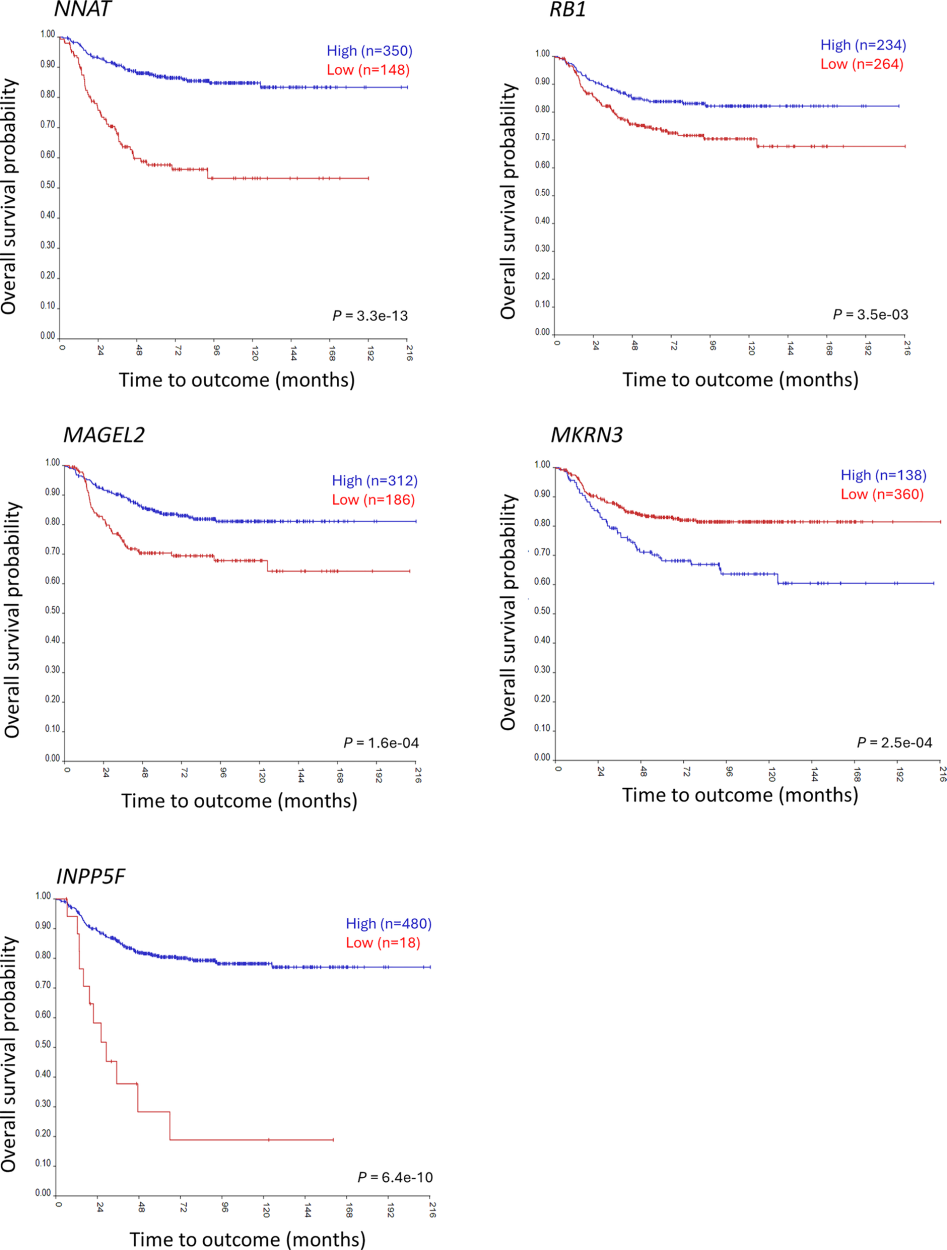
Risk prediction based on gene expression levels. Kaplan–Meier plots generated using the Kaplan scan cutoff method in ‘R2: Genomic Analysis and Visualization Platform’ (http://r2.amc.nl) show the differences in overall survival probabilities in 498 neuroblastoma (NB) samples from publicly available dataset SEQC-498 custom (GSE49710), stratified by gene expression levels of the genes overlapping the imprinted regions

For *MKRN3*, that underwent LOM, a higher gene expression level (theoretically related to a loss in methylation) was found to be associated with poorer overall survival across the three datasets. Whereas for *NNAT*, *RB1* and *INPP5F,* regions that underwent GOM, lower gene expression levels (theoretically related to a gain in methylation) was associated with poorer overall survival. However, at *MAGEL2,* risk stratification based on DNA methylation and gene expression data did not show an expected correlation. Kaplan–Meier plots showing the risk prediction of DMRs based on the gene expression data in the SEQC dataset is presented in Fig. 6.

### Association between DNA methylation and allele-specific CNAs

Chromosomal alterations including whole chromosome gain or loss as well as segmental chromosomal alterations are commonly seen in NB tumours. Gain or loss of a chromosomal segment including or in the vicinity of an imprinted region could also lead to imprinting aberrations. Since data from the methylation arrays are not allele-specific, the deviation from the mono-allelic methylation pattern at the imprinted regions could be both due to epigenetic changes and CNAs, which cannot be distinguished based on only the methylation array data. For instance, both loss of methylation at the methylated allele or a loss of copy number of the methylated allele will result in a low beta-value suggesting a loss of methylation and thus an upregulation in gene expression. However, in reality, in the first situation, there will be an upregulation of gene expression while in the second case the gene expression remains the same as the unmethylated copy remains intact.

So, to understand the relation between CNAs and the observed array-based DNA methylation levels at the imprinted regions, we investigated the allele-specific CNA data (derived using ASCAT from SNP-array data) available for 105 out of the 185 samples included in the TARGET dataset. We combined the allele-specific CNA data and DNA methylation levels at genomic locations mapping to the imprinted DMRs. CNA information was not available for the imprinted region *FANCC*, located on 9q and it was therefore removed from further analysis. Out of 105 NB cases, only three had normal copy number at all imprinted regions, the remaining samples either had a gain or loss of copy number in at least one of the imprinted regions. Fifteen out of 105 (14%) cases had copy number gain (total copy number > 2) in all imprinted DMRs with the most affected regions being *SVOPL* (Chr7:138348774–138348981), *HTR5A* (Chr7:154862770–154863381), *MESTIT1/MEST* (Chr7:130130122–130133110), *SGCE/PEG10* (Chr7:94285642–94287242), *GRB10* (Chr7:50849168–50850870) and PPIEL:Ex1-DMR (Chr1:40024971–40025415).

**Fig. 7:**
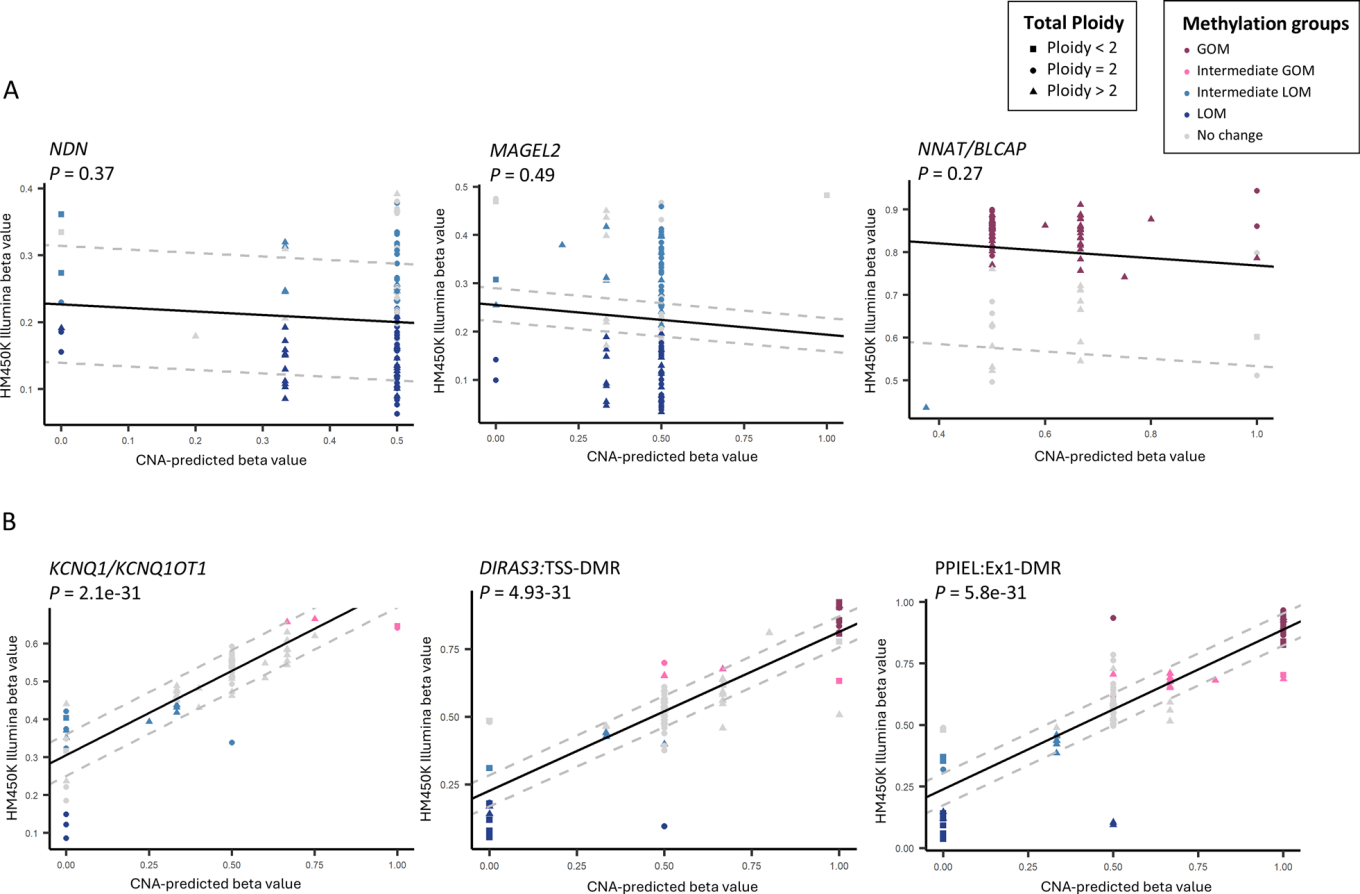
Analysis of copy number alterations and methylation at the imprinted regions. The observed (Illumina Human Methylation 450 k array-based methylation levels) versus expected (copy number alterations (CNAs) based predicted methylation levels) methylation profile of 105 neuroblastoma (NB) tumours in Therapeutically Applicable Research to Generate Effective Treatments (TARGET) dataset at **A** imprinted regions where CNAs were not able to predict the methylation profile as observed based on the methylation array and **B** imprinted DMRs where CNAs were able to predict the methylation profile of NB tumours as observed based on the methylation array. The dashed lines represent the ± 2 standard deviation (SD) of the mean of normal adrenal control tissues. Colours of the data points represent the methylation alteration that the NB tumours display at the imprinted region, which are gain of methylation (GOM) or loss of methylation (LOM), if the absolute beta-value of the NB tumour is above 0.8 or below 0.2, respectively and Intermediate GOM or Intermediate LOM, if the methylation level of NB sample is above or below the healthy controls’ mean 2 SD, respectively. Shape of the data points represent total ploidy of the NB tumours at the imprinted regions. P-value < 0.05 represents significant correlation between the two variables

To further understand the relationship between CNAs and DNA methylation, a prediction of methylation levels based on the allele-specific CNAs was done (as described in the method section). We performed a linear regression analysis with array-based methylation levels as outcome and CNA-based predicted methylation value as predictor. CNAs explained over 50% of the methylation variations observed across several imprinted DMRs (Table 4). Higher adjusted r-squared suggests a higher proportion of observed methylation changes explained by CNAs. However, we found that the regions that had the most altered methylation patterns were not dictated by the underlying CNAs suggesting true epimutations including the regions that underwent most LOM: *NDN* (adjusted R^2^ = − 0.002), *MAGEL2* (adjusted R^2^ = − 0.005), *HTR5A* (adjusted R^2^ = − 0.009), SNRPN:Int1-DMR2 (adjusted R^2^ = − 0.006) and IGF2:Ex9-DMR (adjusted R^2^ = − 0.009) and the regions that underwent most GOM: *NNAT/BLCAP* (adjusted R^2^ = 0.002), *RB1* (adjusted R^2^ = − 0.005) and *GPR1* (adjusted R^2^ = 0.05). For many of these regions the value of adjusted R^2^ was negative indicating that CNAs were not at all a good predictor variable for the observed methylation changes in these regions. Regression plots demonstrating the correlation between observed (HM450K) and CNA-based predicted methylation values for *NDN, MAGEL2* (where no correlation was observed) and for regions where strong correlation was observed as a contrast is presented in Fig. 7A and B, respectively.

**Table 4:** Proportion of methylation variance explained by copy-number alterations

Imprinted DMRs	Adjusted R^2^	*P-value*
KCNQ1OT1:TSS-DMR	0.73532217	2.01E-31
DIRAS3:TSS-DMR	0.72717398	4.94E-31
PPIEL:Ex1-DMR	0.72629062	5.83E-31
GNAS A/B:TSS-DMR	0.68381104	1.81E-27
INPP5F:Int2-DMR	0.67455518	7.93E-27
GNAS-NESP:TSS-DMR	0.65934637	8.24E-26
GNAS-XL:Ex1-DMR	0.63036721	5.41E-24
DIRAS3:Ex2-DMR	0.61564651	2.47E-23
L3MBTL1:alt-TSS-DMR	0.58608032	1.80E-21
MEG3:TSS-DMR	0.55847104	1.13E-19
PLAGL1:alt-TSS-DMR	0.55002662	8.76E-20
PEG10:TSS-DMR	0.54929279	9.53E-20
PEG13:TSS-DMR	0.54917723	9.66E-20
ZNF331:alt-TSS-DMR2	0.53461125	7.41E-19
MEST:alt-TSS-DMR	0.51018985	7.16E-18
GNAS-AS1:TSS-DMR	0.49100914	7.43E-17
WRB:alt-TSS-DMR	0.42274926	8.54E-14
MCTS2P:TSS-DMR	0.42075918	5.83E-14
ZNF331:alt-TSS-DMR1	0.41756253	7.75E-14
PEG3:TSS-DMR	0.41421214	1.04E-13
SNU13:alt-TSS-DMR	0.35982975	8.20E-12
IGF2:alt-TSS-DMR	0.33361469	8.26E-11
IGF1R:Int2-DMR	0.29620759	1.41E-09
WDR27:Int13-DMR	0.25058316	3.23E-08
H19/IGF2:IG-DMR	0.24516293	5.47E-08
MEG8:Int2-DMR	0.2296524	2.08E-07
FAM50B:TSS-DMR	0.2196904	2.75E-07
VTRNA2-1:DMR	0.17697779	4.70E-06
IGF2R:Int2-DMR	0.16691076	9.01E-06
ZDBF2/GPR1:IG-DMR	0.16528803	1.00E-05
SNURF:TSS-DMR	0.16486644	1.13E-05
ERLIN2:Int6-DMR	0.14249812	4.27E-05
MKRN3:TSS-DMR	0.12079245	1.77E-04
GRB10:alt-TSS-DMR	0.0975089	6.93E-04
SNRPN:alt-TSS-DMR	0.07896148	2.24E-03
ZNF597:TSS-DMR	0.05618441	9.11E-03
GPR1-AS:TSS-DMR	0.05523454	9.04E-03
NAP1L5:TSS-DMR	0.05124134	1.26E-02
SVOPL:alt-TSS-DMR	0.03792309	2.60E-02
ZNF597:3' DMR	0.03056095	4.26E-02
SNRPN:Int1-DMR1	0.01495803	1.12E-01
NNAT:TSS-DMR	0.00195492	2.76E-01
NDN:TSS-DMR	− 0.00200942	3.75E-01
RB1:Int2-DMR	− 0.00497893	4.88E-01
MAGEL2:TSS-DMR	− 0.0051687	4.94E-01
SNRPN:Int1-DMR2	− 0.00613663	5.43E-01
IGF2:Ex9-DMR	− 0.00891498	7.65E-01
HTR5A:TSS-DMR	− 0.0095237	8.91E-01

*DMR* Differentially methylated region. P-values are for the linear regression analysis

Adjusted R^2^ are from the linear regression model

## Discussion

Using publicly available clinical and DNA methylation data from TARGET and German Neuroblastoma Trial study, and a Swedish cohort of samples, the methylation profiles of totally 369 NB samples in 49 imprinted DMRs were investigated with the aim of identifying regions that were most altered in NB tumours. We demonstrated that loss in methylation was a more common occurrence compared to a gain in methylation event in NB tumours at the imprinted regions. Key genes overlapping the most altered regions were *NDN*, *SNRPN*, *MAGEL2*, *HTR5A*, *IGF2* and *INS-IGF2* (LOM in > 30% of NB samples) and *NNAT*, *BLCAP*, *RB1*, *GPR1* (GOM in > 30% of NB samples). These regions remained consistent in the validation set, particularly the regions that displayed the most frequent alterations. Many of these genes have previously been reported in cancer and also in the context of NB including *NNAT*, *RB1*, *MAGEL2*, *GPR1, NDN* [38–41]. *NDN,* where LOM was observed in 50% and intermediate LOM in 26% of NB samples, is involved in permanent growth arrest in post-mitotic neurons during the nervous system development [42] and is downregulated in several cancers [40, 41, 43]. Studies show that *NDN* functionally interacts with the p53 protein [44, 45] and have an antagonistic effect on p53-mediated apoptosis [44]. LOI of the IGF2 locus has been implicated in other malignancies. Here we found that IGF2:Ex9-DMR, overlapping *IGF2* and *INS-IGF2* genes, displayed LOM in 41% of NB samples. *MAGEL2,* where 37% NB tumours displayed LOM and 28% Intermediate LOM, belongs to a highly conserved group of proteins (MAGE-The Melanoma Antigen Gene), which are reported to be deregulated in multiple cancers [46–48] including central nervous system tumours medulloblastoma [49] and glioblastoma [50]. *NNAT* that underwent GOM in a larger proportion of NB tumours (75%) is involved in mammalian brain development and has previously been reported in NB as a potential tumour suppressor [38]. High expression levels of *NNAT* have been found to be associated with good prognosis [38]. A recent study reported the involvement of *NNAT* with oxidative stress in ER + breast cancer [51]. This is an interesting link as oxidative stress is also reported in NB [52] and it may be related to the deregulation in *NNAT* gene expression. *RB1* overlapping with the imprinted region RB1:Int2-DMR, where 46% of NB tumours displayed GOM in our study, has also previously been reported as an independent prognostic biomarker for NB where low *RB1* expression correlated with poor prognosis [39]. Our data also illustrated that several of the most altered regions in NB showed correlations with aggressive tumour behavior that included older age at diagnosis, higher stage tumour and amplification of *MYCN*. The strongest correlation was observed for *NNAT/BLACP* and *RB1* and *MAGEL2* and the tumours that underwent methylation alterations at these regions were > 1.5 years of age, had stage 4 tumour and were *MYCN* amplified. A correlation between the total number of imprinted DMRs undergoing methylation changes and clinical behaviour was also observed (Fig. 3). Patients that accumulated methylation changes in a larger number of imprinted regions were diagnosed at an older age (above 1.5 years), with more advanced tumour (stage 3 and stage 4) and with an amplification of *MYCN.* Interestingly, NB cases with 11q deletion did not differ significantly in terms of number of imprinted regions undergoing methylation changes with the cases that had no alteration at 11q. On the other hand, cases that had loss of whole chromosome 11 had significantly lesser number of imprinted regions with methylation alterations. This is consistent with previous reports where loss of whole chromosomes were found to be associated with less aggressive clinical behaviour and better prognosis [10]. Also consistent with previous findings where *TMM negative* molecular subclass has been associated with better prognosis [37], we found that the NB cases in this subclass had the lowest number of imprinted regions with altered methylation patterns. In contrast, the *MYCN Type* had the largest number of imprinted regions with altered methylation patterns. These results suggest that alterations in methylation patterns at imprinted regions has a role in promoting tumour growth, and accumulation in terms of number is also indicative of more aggressive clinical features.

Imprinting dysregulation has been shown to be associated with tumour progression and patient survival [24]. We tested the association between methylation alterations at the 49 imprinted DMRs and overall survival probabilities of patients. Our data showed that methylation alterations at six of the 49 imprinted DMRs tested was associated with shorter overall survival (*MIR886*, *RB1*, *NNAT/BLCAP*, *MAGEL2*, *MKRN3* and *INPP5F*). GOM at *NNAT/BLCAP* (*P* = 8.1e-05), *RB1* (*P* = 0.005), *MIR886* (P = 0.0002) and *INPP5F* (GOM, *P* = 0.01) correlated significantly with poorer overall survival. A gain of methylation (GOM) could be hypothesized to result in reduced expression of the corresponding gene. Through gene expression-based stratification of patients in the R2 database, we demonstrated that for the genes mapping to the regions i.e. *NNAT*, *RB1* and *INPP5F*, that underwent GOM, a lower gene expression (related to higher DNA methylation levels) was associated with shorter survival. Gene expression-based prognostication could not be tested for *MIR886* as data was not available. However, gain of methylation at this gene has previously been reported to be predictive of poor prognosis in lung cancer [53], esophageal cancer [54] and in leukemia [55]. LOM at *MKRN3* (*P* = 0.04) and *MAGEL2* (*P* = 0.0001) correlated significantly with poorer overall survival. We found that higher gene expression level (related to a loss of methylation) was associated with poorer overall survival for *MKRN3* in the R2 database*.* This therefore indicated that the imprinted genes could stratify patients into risk group based on the DNA methylation levels that also relates to the gene expression levels.

Amplification of *MYCN* and deletion at 11q are established prognostic markers for NB with poor outcome, where *MYCN* amplification is present in 20–30% [56] and 11q deletion is present in 35–45% of all NB tumours [10]. These genetic events are rarely detected in a single tumour [57, 58]. Our data further demonstrated that methylation changes at *RB1*, *NNAT/BLCAP* and *MKRN3* were able to more precisely and further sub-stratify *MYCN* non-amplified and 11q normal cases (cases lacking amplification of *MYCN* and 11q deletion) into risk groups, which is currently treated as one low-risk group based on the existing risk markers. Among patients lacking *MYCN* amplification and 11q deletion, methylation changes at these sites were significantly associated with poorer prognosis when compared with the patients with no alterations in methylation at these sites. This suggests that among the patient group without *MYCN* amplification and 11q deletion, currently treated as low-risk, there are sub-groups with increased risk that are missed. Our data demonstrated that cases with 11q-deletion and absence of *MYCN* amplification which also harbored methylation changes at *RB1*, *NNAT/BLCAP* and *MKRN3* had the poorest survival probability (Fig. 4B). This suggest that incorporating methylation status of the imprinted regions with the existing risk predictors will make the prognostication more precise. This was further supported by the multivariate analysis where we compared the prognostic significance of the imprinted genes, adjusting for age at diagnosis, *MYCN* amplification or 11q deletion. We demonstrated that methylation alterations at *NNAT/BLCAP* (*P* = 0.03) and *MAGEL2* (*P* = 0.01) predicted worse outcome independently of age at diagnosis and *MYCN* amplification. While methylation alterations at *MIR886* show a slightly weaker association with increased hazard in *MYCN* amplified NB tumours (Fig. 5A), methylation alterations at this region were found to be strongly associated with increased hazard in NB tumours with 11q deletion (Fig. 5B).

Ribaraska et al., (2014) reported several imprinted genes with deregulated gene expression in prostate cancer but they displayed a stable DNA methylation pattern, suggesting that somatically acquired CNAs in the close vicinity of imprinted genes may also be the cause of LOI [40]. This finding was supported by another recent study that showed that methylation profiles at imprinted DMRs largely represent the accumulation of CNAs [31]. Using matching allele-specific CNAs data from the TARGET dataset, we demonstrated that the regions that displayed most alterations in methylation patterns were found to be true epigenetic changes and were not due to CNAs in their vicinity. However, CNAs do dictate the observed methylation alterations in many of the imprinted regions. One limitation of the study was that only 49 imprinting regions were investigated and there may be other imprinted regions that are deregulated in NB. In addition, some of the imprinted DMRs contained few CpG probes that may not provide a true representation of methylation pattern in those regions and thus require further validation. Further study using whole-genome bisulfite sequencing is warranted that would offer more precise allele-specific methylation patterns at the identified imprinted regions. Validating the findings across independent datasets would help address the heterogeneity of NB and ensure that the identified methylation patterns are consistent with prognosis. Understanding how these alterations affect gene expression and tumor behaviour could provide deeper insights into their potential as therapeutic targets.

## Conclusions

We demonstrated that imprinting dysregulation is prevalent in NB and methylation alterations at imprinted genes may explain differences in patient prognosis and can be used as independent prognostic biomarkers. Incorporating methylation status of imprinted regions could refine current prognostication and identify further risk sub-groups within the low-risk group.

### Supplementary Information

Additional file 1

Additional file 2

## Data Availability

The results published here are in part based upon data generated by the Therapeutically Applicable Research to Generate Effective Treatments (https://www.cancer.gov/ccg/research/genome-sequencing/target) initiative, phs000218. The data used for this analysis is available at the Genomic Data Commons (https://portal.gdc.cancer.gov).” Other datasets used in the current study are available from the corresponding author on reasonable request.

## Abbreviations

BH: Benjamini-Hochberg
CNAs: Copy number alterations
CI: Confidence interval
COG: Children’s Oncology Group
DMRs: Differentially methylated regions
GEO: Gene expression omnibus
GOM: Gain of methylation
HR-NB: High-risk neuroblastoma
HM450K: HumanMethylation450
HR: Hazard ratio
INSS: International neuroblastoma staging system
LOI: Loss of imprinting
LOM: Loss of methylation
mUPD: Maternal uniparental diploidy
MNP: Molecular neuropathology
NB: Neuroblastoma
pUPD: Paternal uniparental diploidy
SNP: Single nucleotide polymorphism
SD: Standard deviation
TSS1500: Transcription start site 1500
Tmm: Telomerase maintenance mechanism
TARGET: Therapeutically applicable research to generate effective treatments
5’UTR: 5’ Untranslated region
3’UTR: 3’ Untranslated region
