# Supplementary material for: Altered methylation of imprinted genes in neuroblastoma: implications for prognostic refinement
Source: J Transl Med. 2024 Aug 31;22:808. doi: 10.1186/s12967-024-05634-5 (PMC11366169; doi:10.1186/s12967-024-05634-5)
Supplement: Supplementary file 1 — Additional file 1 [file 12967_2024_5634_MOESM1_ESM.pptx]

## Slide 1
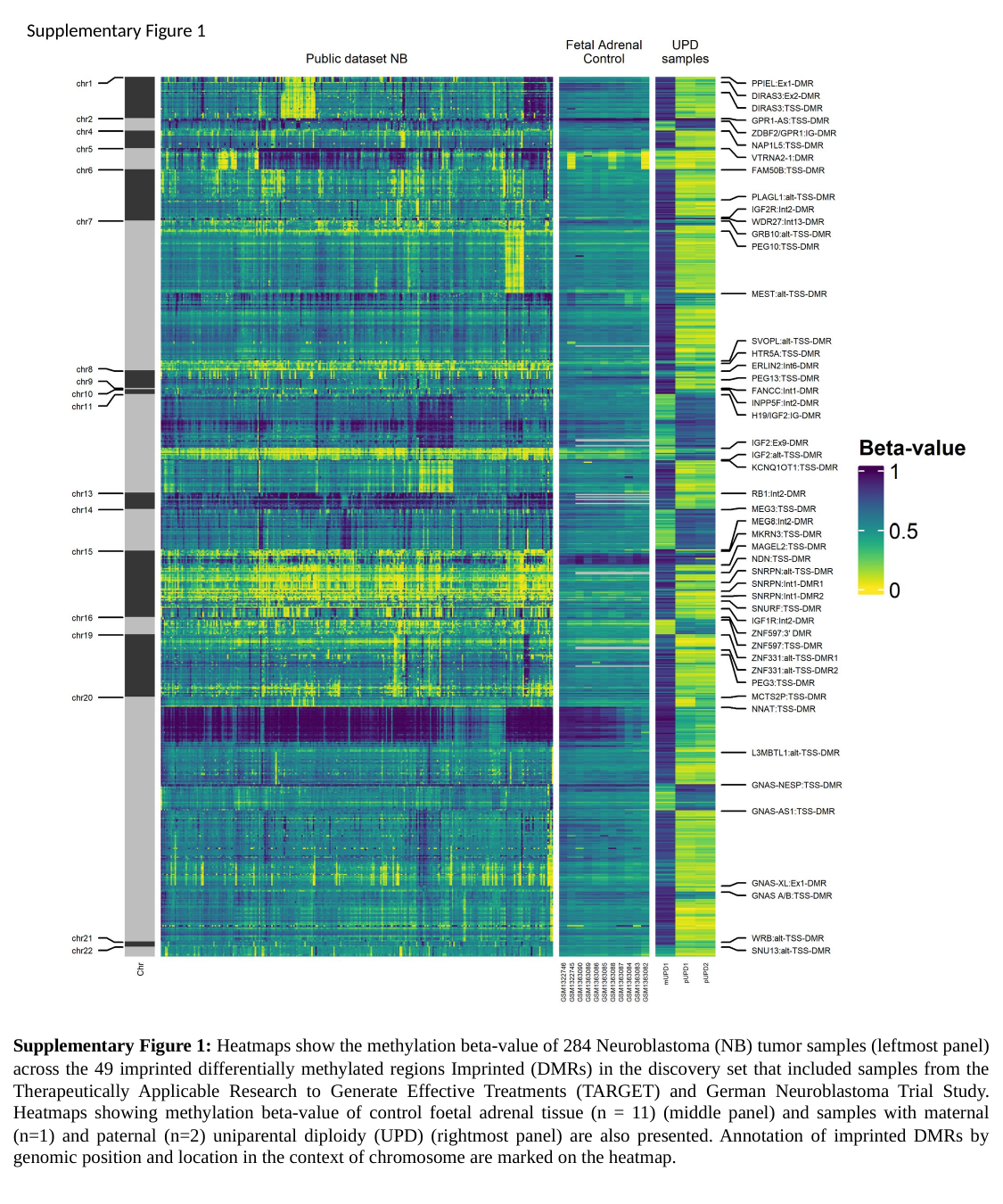

Supplementary Figure 1
Supplementary Figure 1: Heatmaps show the methylation beta-value of 284 Neuroblastoma (NB) tumor samples (leftmost panel) across the 49 imprinted differentially methylated regions Imprinted (DMRs) in the discovery set that included samples from the Therapeutically Applicable Research to Generate Effective Treatments (TARGET) and German Neuroblastoma Trial Study. Heatmaps showing methylation beta-value of control foetal adrenal tissue (n = 11) (middle panel) and samples with maternal (n=1) and paternal (n=2) uniparental diploidy (UPD) (rightmost panel) are also presented. Annotation of imprinted DMRs by genomic position and location in the context of chromosome are marked on the heatmap.

## Slide 2
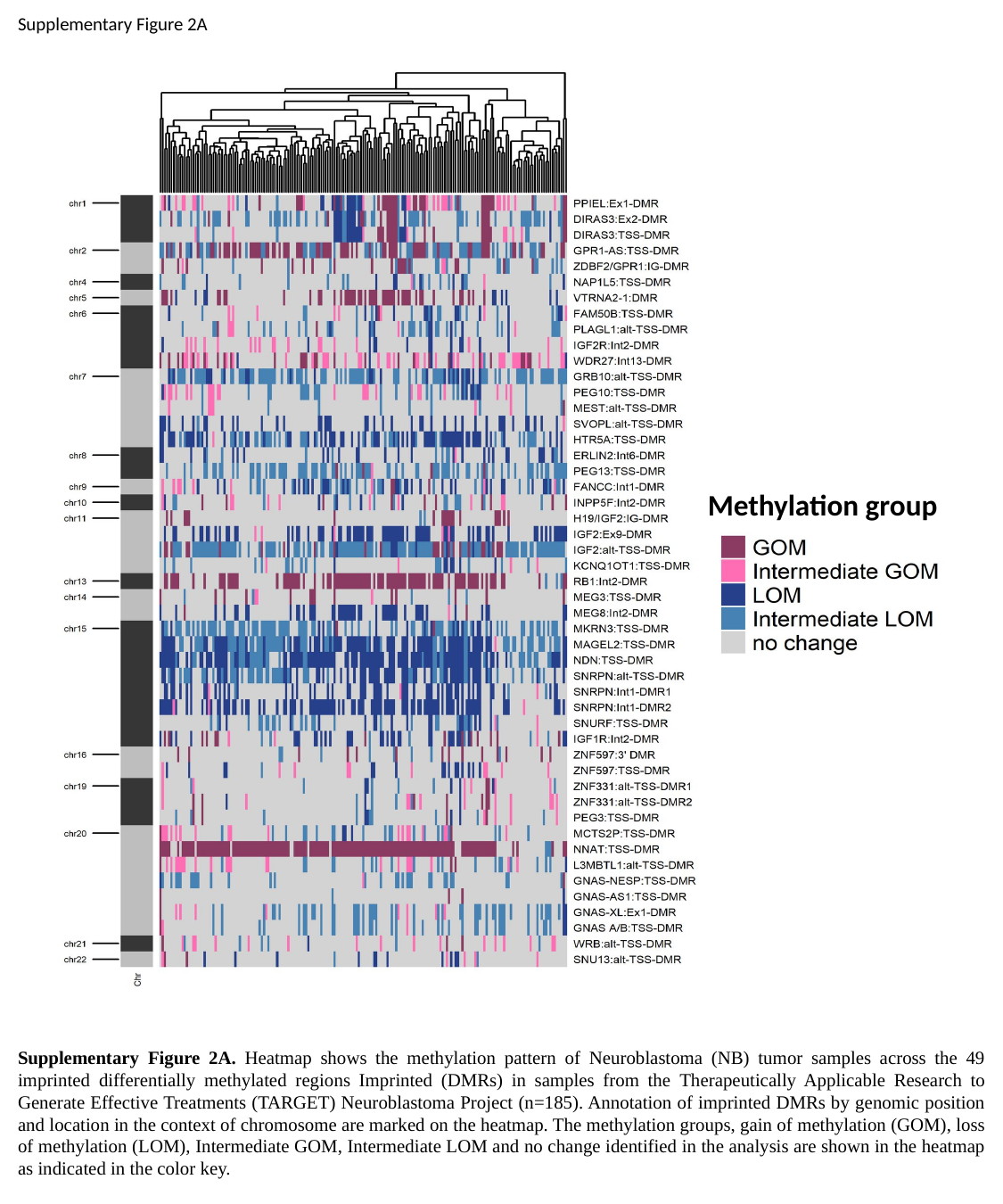

Supplementary Figure 2A
Methylation group
Supplementary Figure 2A. Heatmap shows the methylation pattern of Neuroblastoma (NB) tumor samples across the 49 imprinted differentially methylated regions Imprinted (DMRs) in samples from the Therapeutically Applicable Research to Generate Effective Treatments (TARGET) Neuroblastoma Project (n=185). Annotation of imprinted DMRs by genomic position and location in the context of chromosome are marked on the heatmap. The methylation groups, gain of methylation (GOM), loss of methylation (LOM), Intermediate GOM, Intermediate LOM and no change identified in the analysis are shown in the heatmap as indicated in the color key.

## Slide 3
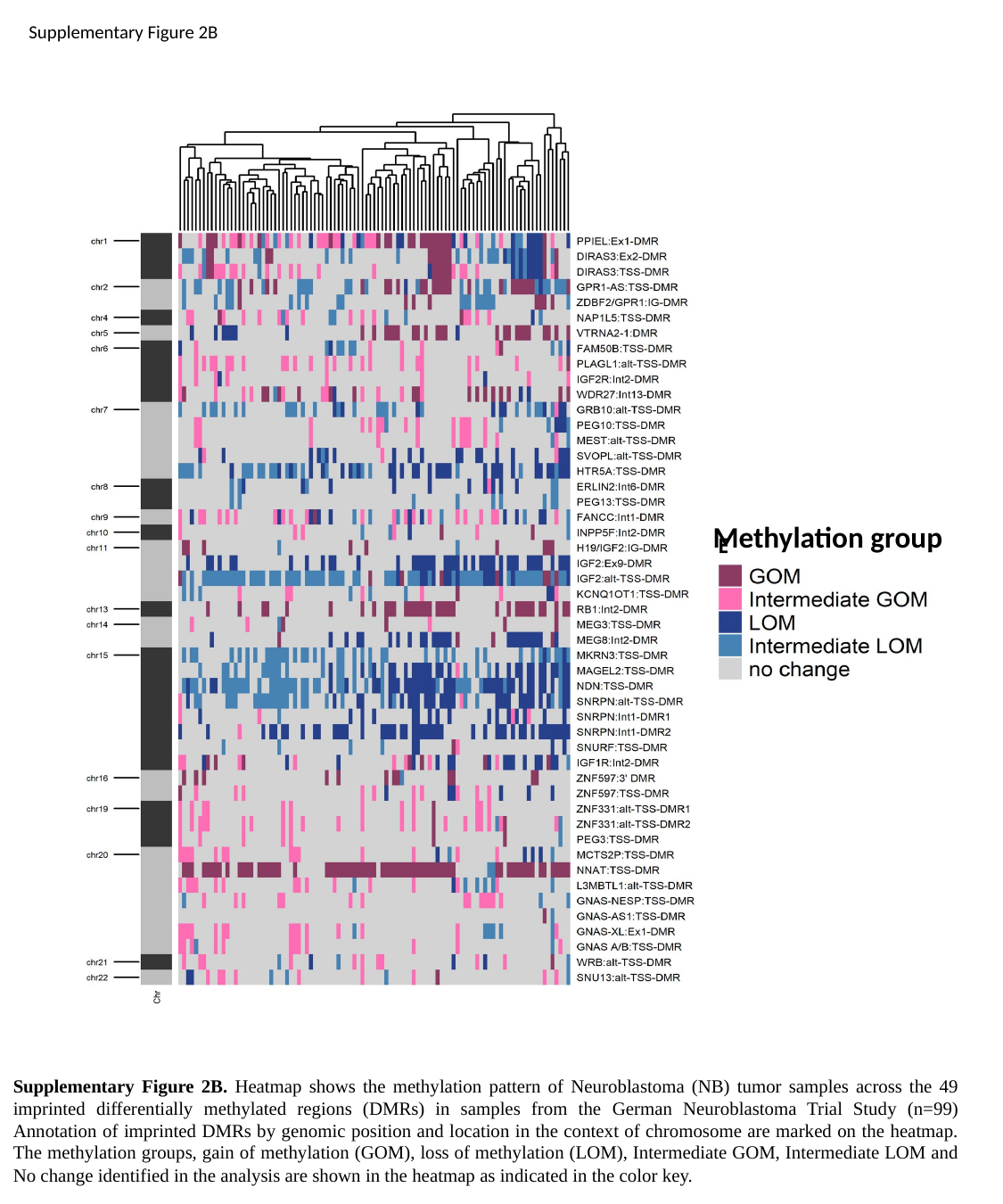

Supplementary Figure 2B
Methylation group
Supplementary Figure 2B. Heatmap shows the methylation pattern of Neuroblastoma (NB) tumor samples across the 49 imprinted differentially methylated regions (DMRs) in samples from the German Neuroblastoma Trial Study (n=99) Annotation of imprinted DMRs by genomic position and location in the context of chromosome are marked on the heatmap. The methylation groups, gain of methylation (GOM), loss of methylation (LOM), Intermediate GOM, Intermediate LOM and No change identified in the analysis are shown in the heatmap as indicated in the color key.

## Slide 4
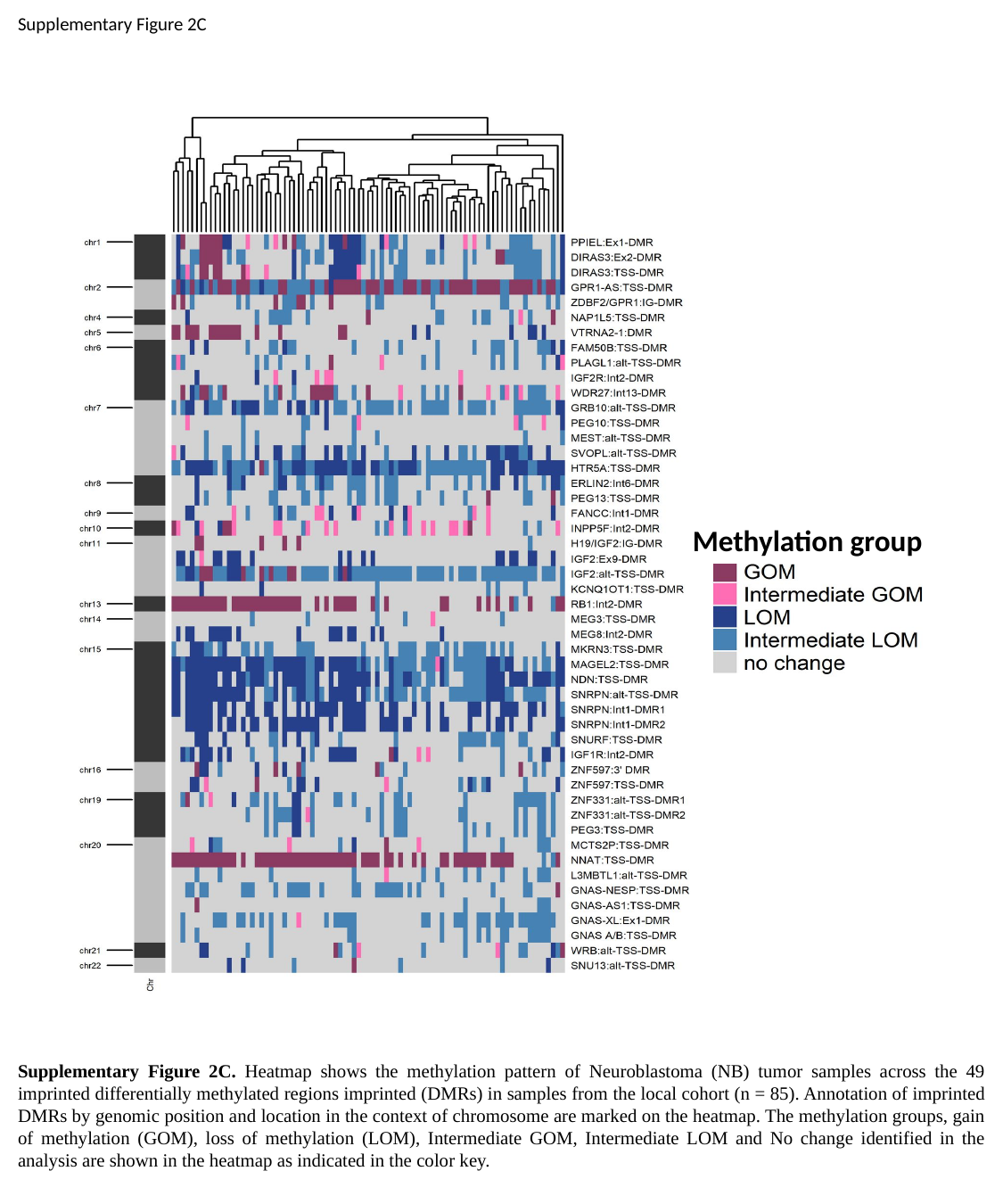

Supplementary Figure 2C
Methylation group
Supplementary Figure 2C. Heatmap shows the methylation pattern of Neuroblastoma (NB) tumor samples across the 49 imprinted differentially methylated regions imprinted (DMRs) in samples from the local cohort (n = 85). Annotation of imprinted DMRs by genomic position and location in the context of chromosome are marked on the heatmap. The methylation groups, gain of methylation (GOM), loss of methylation (LOM), Intermediate GOM, Intermediate LOM and No change identified in the analysis are shown in the heatmap as indicated in the color key.

## Slide 5
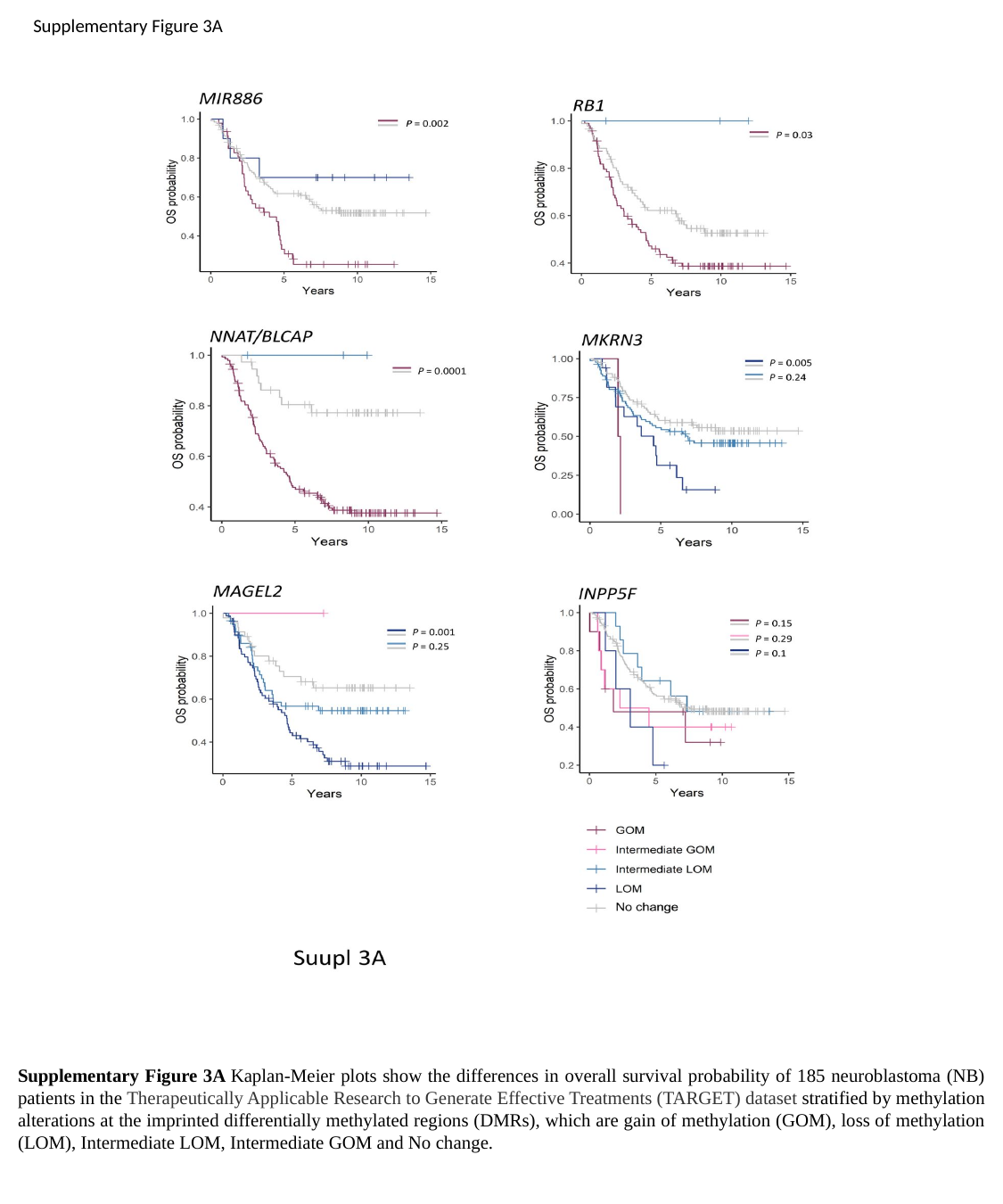

Supplementary Figure 3A
Supplementary Figure 3A Kaplan-Meier plots show the differences in overall survival probability of 185 neuroblastoma (NB) patients in the Therapeutically Applicable Research to Generate Effective Treatments (TARGET) dataset stratified by methylation alterations at the imprinted differentially methylated regions (DMRs), which are gain of methylation (GOM), loss of methylation (LOM), Intermediate LOM, Intermediate GOM and No change.

## Slide 6
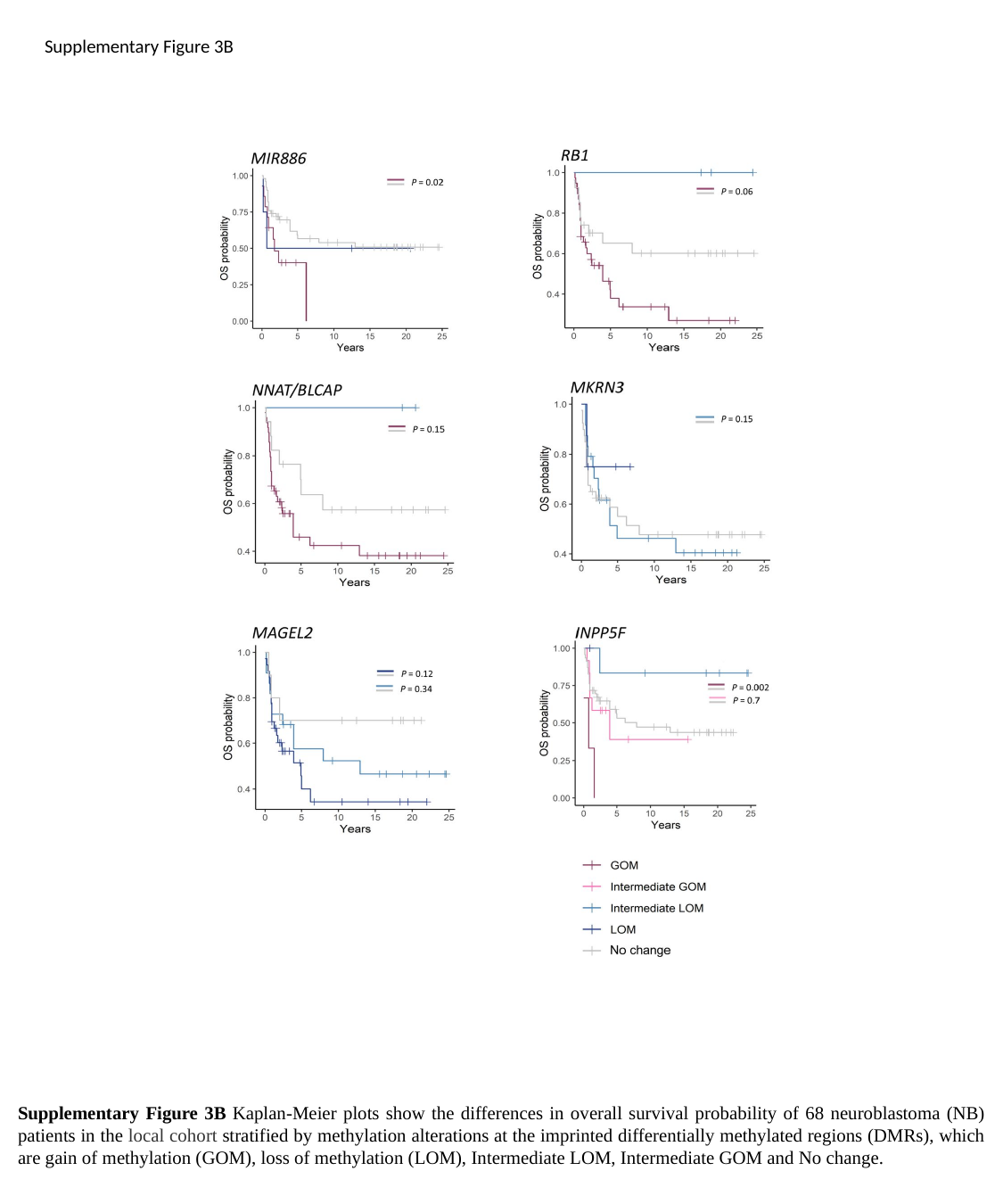

Supplementary Figure 3B
Supplementary Figure 3B Kaplan-Meier plots show the differences in overall survival probability of 68 neuroblastoma (NB) patients in the local cohort stratified by methylation alterations at the imprinted differentially methylated regions (DMRs), which are gain of methylation (GOM), loss of methylation (LOM), Intermediate LOM, Intermediate GOM and No change.
